# PLCG2 can exist in eccDNA and contribute to the metastasis of non-small cell lung cancer by regulating mitochondrial respiration

**DOI:** 10.1038/s41419-023-05755-7

**Published:** 2023-04-08

**Authors:** Yongfeng Yang, Ying Yang, Hong Huang, Tingting Song, Shengqiang Mao, Dan Liu, Li Zhang, Weimin Li

**Affiliations:** 1 Department of Respiratory and Critical Care Medicine, Institute of Respiratory Health, Center of Precision Medicine, West China Hospital, Sichuan University, Chengdu, Sichuan Province China; 2 Institute of Clinical Pathology, Key Laboratory of Transplantation Engineering and Immunology, Ministry of Health, West China Hospital, Sichuan University, Chengdu, Sichuan Province China

**Keywords:** Non-small-cell lung cancer, Cancer metabolism

## Abstract

Extrachromosomal circular DNAs (eccDNAs) participate in tumorigenesis and tumor progression. However, the role and mechanism of eccDNAs have yet to be elucidated in non-small cell lung cancer (NSCLC). In our research, three surgically matched NSCLC tissue samples, NSCLC cell lines (H1299, A549, and H460), and a normal lung cell line (MRC-5) were used as study objects. High-throughput eccDNA sequencing and bioinformatics analysis were performed to study the distribution pattern and level of eccDNA expression. The upregulated candidate eccDNA-encoding PLCG2 was validated by routine PCR. Plasmid transfection, RNA interference, qRT‒PCR and western blotting experiments were used to verify the expression level of PLCG2. Our results showed that the chromosome distribution, length distribution, and genomic annotation of the eccDNAs were comparable between the NSCLC and normal groups. Nevertheless, there were no significant differences in eccDNAs between NSCLC tissues and matched normal lung tissues. The eccDNA derived from PLCG2 was upregulated in NSCLC cells. TCGA analysis and immunohistochemistry showed that PLCG2 was highly expressed in lung cancer tissues and tended to be associated with poor outcome. We also demonstrated that PLCG2 can promote metastasis through the regulation of mitochondrial respiration. These results suggested that PLCG2 identified by eccDNA sequencing acts as an oncogene and might be a new biomarker for NSCLC diagnosis and prognosis evaluation.

## Introduction

Lung cancer is the leading cause of cancer-related death, accounting for approximately 350 deaths per day [1]. Non-small cell lung cancer (NSCLC) accounts for 80% of all lung cancer cases and remains the dominant pathological type [2]. Despite rapid advances in the diagnosis and treatment of lung cancer, the overall 5-year survival rate remains poor [3]. Elucidating the molecular mechanisms underlying the high incidence and poor prognosis of NSCLC would be helpful for developing effective therapies.

Although the majority of human cellular DNA is linear chromosomal DNA, genes can also be found outside of chromosomes [4]. Most extrachromosomal genes are extrachromosomal circular DNA (eccDNAs), which have a closed circular configuration and are commonly observed in both normal and cancer cells [5–7]. The size of eccDNAs varies from hundreds of base pairs (bp) to several megabases (Mb) [4]. Large eccDNAs (ecDNAs) include the entire spectrum of large, gene-containing extrachromosomal particles, such as double minutes (DMs), and participate in cancer initiation by carrying oncogenes and promoting gene amplification [8, 9]. However, the majority of eccDNAs in mammalian tissues and cell lines are too small to carry protein-coding genes; these small eccDNAs include small polydispersed DNAs (spcDNAs) (100 bp–10 kb), telomeric circles (t-circles) (multiples of 738 bp), and microDNAs (100 bp–400 bp) [10], which are associated with genomic instability and may modulate gene expression through the production of both known and novel regulatory small RNAs [7, 11]. We will use eccDNAs in the following text as a term for the full range of circular DNAs found in eukaryotes.

There is growing evidence that eccDNAs are associated with cancer progression [12]. EccDNAs commonly harbor oncogenes or drug resistance genes and thus participate in the stress response to exogenous and endogenous stimuli, aging, carcinogenesis and drug resistance during cancer treatment [9, 13, 14]. An improved understanding of the functional role of eccDNAs in cancer would provide insight into the complex biology of cancer and open up new avenues for cancer research. In addition, clarification of the unique topological structure and genetic characteristics of eccDNAs could provide ideas for cancer monitoring, early diagnosis, treatment, and prognosis prediction and provide novel biomarkers for cancer [15, 16]. High-throughput sequencing demonstrated that the size distribution of eccDNAs varied between maternal and fetal plasma, which suggested that eccDNAs are potential markers for distinguishing different biological groups [17]. The eccDNAs identified in lung cancer samples were longer than those in matched normal tissues, and the size of circulating eccDNAs in the plasma decreased after surgical resection of lung cancer [15]. The key question regarding the value of eccDNA as a biomarker for cancer is whether tumor-generated eccDNAs can be distinguished from those produced in normal tissue [18–22].

Phospholipase C gamma 2 (PLCG2) is a transmembrane signaling enzyme that is involved in transmitting signals from growth factor receptors and immune system receptors across the cell membrane [23]. PLCG2 has been shown to be involved in tumor microenvironment (TME) remodeling and has been identified as an immune-related gene in soft tissue sarcomas and colon cancer [24, 25]. PLCG2 expression in a subpopulation of cells has been shown to confer the aggressiveness seen in SCLC and a subpopulation of cells with a high expression of PLCG2; this malignant phenotype is also represented by the enrichment of genes related to metastasis, chemotaxis, and stemness, including genes involved in WNT and BMT signaling and EMT [26].

In this study, we first investigated the different expression profiles of eccDNAs between NSCLC samples and normal lung samples (including tissues and cells). We confirmed that eccDNAs are derived from every human chromosome with sequences from all known types of genomic structures, including genes and intergenic and repetitive regions, thus revealing that eccDNAs are common mutational elements in NSCLC. GO and KEGG analyses revealed that eccDNAs were enriched in cancer-related pathways, including oxidative phosphorylation, mitochondrial respiration and the NF-kappa B signaling pathway. Finally, we compared the eccDNA profiles between NSCLC samples and normal lung samples. Although there were no differentially expressed eccDNAs between NSCLC and matched normal lung tissues, we identified that PLCG2 eccDNA was upregulated in NSCLC cells, and we confirmed that PLCG2 had high expression in NSCLC tissues and cells. Thus, we indicated that PLCG2 can exist in the form of eccDNA in lung cancer and found that PLCG2 could promote the metastasis of NSCLC cells by enhancing mitochondrial function as an oncogene. Our findings suggested that upregulated genes present as eccDNA may be oncogenes in NSCLC and play vital roles in carcinogenesis. Identification and clarification of highly expressed genes in the form of eccDNA and their mechanisms may provide new strategies for NSCLC therapy.

## Materials and methods

### Cell culture, transfection and tissue samples

This study was approved by the Ethics Committee at West China Hospital, Sichuan University [IRS number: 2021680A], China, in accordance with the ethical guidelines of the Declaration of Helsinki (as revised in 2013). Five human NSCLC cell lines (H1299 RRID:CVCL_0060, A549 RRID:CVCL_0023, H460 RRID:CVCL_0459, H1975 RRID:CVCL_1511, and PC9 RRID:CVCL_DI28), and MRC-5 human embryonic lung fibroblasts (RRID:CVCL_JF54) were purchased, and these cells were verified by short tandem repeat (STR) genotyping from Guangzhou Cellcook Biotech Co. (Guangzhou, China), and the test for mycoplasma contamination was negative. NSCLC cells were cultured in RPMI 1640 medium (Thermo Fisher Scientific), MRC-5 cells were grown in Dulbecco’s modified Eagle’s medium (DMEM), supplemented with 10% fetal bovine serum (FBS; Gibco), 100 units/mL penicillin, and 0.1 mg/mL streptomycin at 37 °C in the presence of 5% CO^2^. The siRNA and the overexpression plasmid of PLCG2 with their negative control (vector) were synthesized by GeneCopoeia, and the transfections were performed with Lipo3000 reagent (Invitrogen, USA).

Three pairs of tumor tissue samples and matched nontumor lung tissues (male age 45, male age 54, and female age 64 years) were obtained during surgery in the center of lung cancer at West China Hospital, and the nontumor tissues were collected from the distal end of the matched lung cancer. Informed consent was obtained from each patient.

### EccDNA enrichment and sequencing

High-throughput eccDNA sequencing and subsequent bioinformatics analyses were performed by Genedenovo Biotech Inc. (Guangzhou, China). EccDNA enrichment, purification and sequencing were performed as described in a previous study [27]. Sequencing was carried out on an Illumina NovaSeq 6000 with 150 bp paired-end mode according to the manufacturer’s instructions.

### Validation of eccDNA by routine polymerase chain reaction (PCR)

Experimental validation was performed to detect the upregulated eccDNA, which encoded PLCG2 in H1299 cells, as described in a previous study [28]. PCR products were loaded onto 2% agarose gels and visualized under an ultraviolet Luminescent Image Analyzer (LAS-4000 Mini; GE Healthcare Life Sciences, Pittsburgh, USA).

### RNA extraction and quantitative real-time PCR

TRIzol reagent (Invitrogen, CA, USA) was used to extract total RNA from cells and tissues. The reaction conditions and quantitative real-time PCR system were operated according to the kit instructions. The primer sequences are listed in Supplementary Table S8.

### Western blotting

Cultured cells or tissues were lysed in RIPA buffer with 1% PMSF. Protein was loaded onto an SDS‒PAGE minigel and transferred onto a PVDF membrane. After being probed with primary antibodies at 4 °C overnight, the blots were incubated with secondary antibody. Signals were visualized using ECL Substrates (Millipore, MA, USA). GAPDH was used as an endogenous protein for normalization. The antibodies are listed in Supplementary Table S9.

### Immunohistochemistry

Human NSCLC tumor tissues were fixed with 4% paraformaldehyde for 4 h at room temperature, and paraffin sections (5-μm thick) were used for IHC analysis. The sections were processed and stained with PLCG2 antibody.

### Cell proliferation, migration, invasion and colony formation assays

Cell proliferation, migration and invasion were measured using Cell Counting kit-8 (CCK-8) (Beyotime, Hangzhou, China) assays and the transwell system (8-μm pore size, Millipore, USA) according to the kit instructions.

In the clonogenic assay, cells were suspended and plated at a density of 600 cells per 34.8 mm dish. Two weeks later, all formed colonies were stained with 0.05% crystal violet for 30 min. The assays were performed in duplicate plates, and a total of nine fields were counted for each plate.

In the wound healing assay, cells were plated at a proper density in 6-well dishes and scratched with pipette tips. Microscopy was used to detect changes in the scratch area after 0, 24 and 48 h.

### Flow cytometric assays of cell cycle and apoptosis

The Cell Cycle Detection kit and Annexin V Apoptosis Detection kit (Life Technologies, Grand Island, NY) were used to assess the cell cycle distribution and apoptosis rate according to manufacturer’s instructions. All analyses were performed with FlowJo software (Tree Star Inc.).

### Glucose uptake, pyruvate, lactate, and cholesterol detection

A glucose uptake detection kit (Biovision), pyruvate assay kit, lactate assay kit, and cholesterol assay kit (Biovision) were used to detect glucose uptake and the levels of pyruvate, lactate, and cholesterol following the manufacturer’s instructions.

### Measurement of ATP and ROS concentration

The ATP and ROS concentrations were measured using the corresponding commercial kits (#S0026 and #S0033S, Beyotime Biotechnology) according to the manufacturer’s instructions. The standard ATP curve was generated by using a series of standards with known concentrations.

### Glycolytic rate and mitochondrial oxygen consumption rate detection

The glycolytic rate and mitochondrial oxygen consumption rate were measured with the Agilent Seahorse XF Glycolytic Rate Assay kit and Agilent Seahorse XF Cell Mito Stress Test kit with an XF24 Analyzer (Seahorse Bioscience, North Billerica, MA) according the manufacturer’s guidelines. The data were normalized to the number of cells present in each well.

### Mitochondrial labeling and observation

MitoTracker Deep Green (Invitrogen, USA) was used to label the mitochondria, which were observed using a confocal microscope (Nikon). Mitochondrial length was measured with ImageJ software.

### Mitochondrial membrane potential (MMP) detection

A JC-1 staining kit (Abcam, UK) was used to detect MMP according to the instructions and the fluorescence intensity was analyzed with flow cytometry (Beckman Coulter, CA, USA).

### Mitochondrial DNA (mtDNA) detection

Human Mitochondrial DNA (mtDNA) Monitoring Primer Set (Takara, #7246) with four primer pairs (ND1, SLCO2B1, ND5, SERPINA1 Primer Mix) was used to quantify the relative number of copies of human mitochondrial DNA (mtDNA) with nuclear DNA (nDNA) content as a standard by real-time PCR according to the manufacturer’s instructions.

### Electron microscopy imaging

The cells were fixed with 3% glutaraldehyde and 1% osmium tetroxide, and after dehydration step by step with acetone, the samples were embedded in Epon 812 resin and cut into 50-nm-thick slices. After staining with uranyl acetate and lead citrate, images were acquired by a JEM-1400 PLUS transmission electron microscope.

### Animal study

Nude mice (6 mice/group with randomization, male, 6–8 weeks old, 18–20 g) were injected intravenously with 10^6^ cells via tail–vein injection [28]. All mice were sacrificed 3 weeks after injection, and lung metastatic nodules were counted with blinding. All experimental procedures involving mice were performed according to the guidelines of the Swiss Federal Veterinary Office and the regulations of the Cantonal Veterinary Office of Basel Stadt (Licenses 1878, 1907, 1908) [29]. This study was approved by the Ethics Committee at West China Hospital, Sichuan University [IRS number: 2021680A], China.

### Statistical methods

The significance of differences for all normally distributed data was reported according to standard significance thresholds as standardized statistical representations. Significance testing was performed with either the two-tailed Student’s t test or one-way ANOVA analysis of variance. Wilcoxon rank-sum tests (for two groups) were applied to determine confidence intervals. Nonparametric Wilcoxon rank-sum tests (for two groups) were also performed to confirm the results. The correlation between the ratio of coding genes/Mb and eccDNA/Mb in any of the chromosomes was performed by linear regression analysis. Differences in |LogFC| > 2, *P* value < 0.05 were used for the standard screening of differentially expressed genes. The Cluster Profile software was used for GO functional enrichment analysis of the differential gene set. KOBAS 2.0 software was used to screen the differential gene enrichment into the signal pathway in KEGG database, and *P* < 0.05 was used as the threshold of difference significance. All experiments were replicated three times, and the values were averaged. All statistical analyses were performed using GraphPad Prism 8.0 (GraphPad Software, San Diego, CA, USA). Alpha was set at 0.05, and all tests were two-tailed. Data were analyzed with the SPSS 17.0 statistical software program. Results were expressed as “mean ± standard deviation”, and *P* < 0.05 indicated significant difference.

## Results

### Genome-wide detection and analysis of eccDNAs in matched NSCLC tissue

**Fig. 1:**
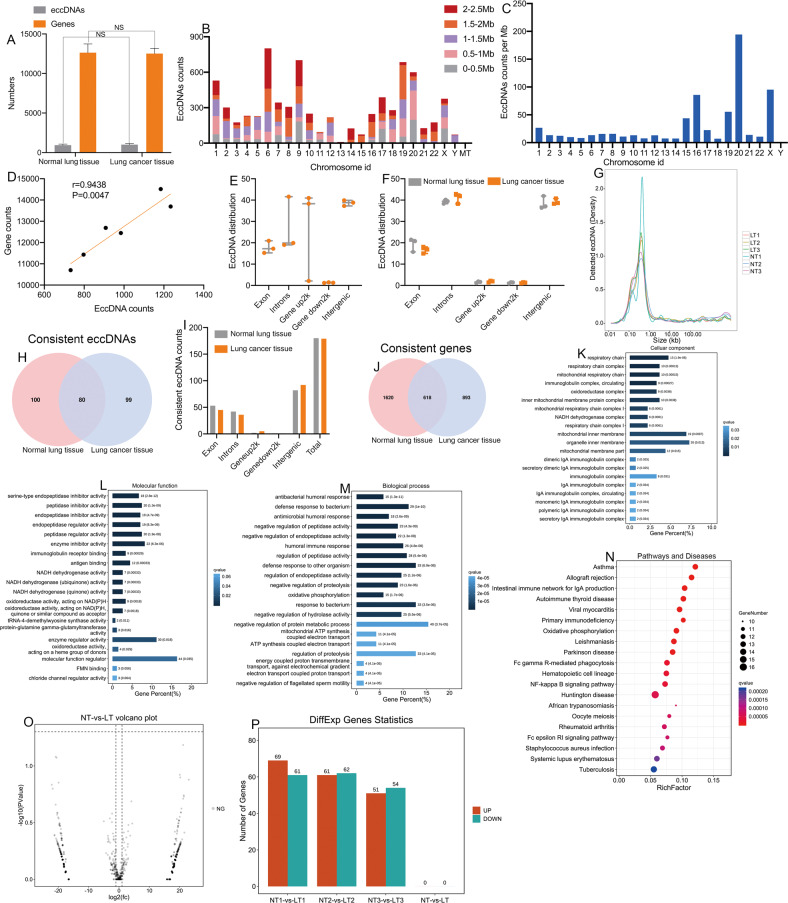
Genome-wide detection and analysis of eccDNA distribution by high-throughput sequencing in matched NSCLC and normal samples. **A** The number of eccDNA types and involved genes from normal and NSCLC tissues. **B** The distribution of eccDNAs in the 23 pairs of chromosomes. **C** The eccDNA frequency per Mb in each chromosome. **D** There was a significant correlation between the number of coding genes in a given chromosome and the number of eccDNAs derived from the chromosome (*P* = 0.047). **E**, **F** The distribution of eccDNAs in different classes of genomic regions. **G** The length distribution of eccDNAs ranged from 0.01 kb to 1000 kb. **H** A Venn diagram showing the consistent eccDNAs detected in the NSCLC and matched normal lung tissue samples. **I** The distribution of consistent eccDNAs in different classes of genomic regions. **J** A Venn diagram showing the consistent genes detected in the NSCLC and matched normal lung tissue samples. **K**–**M** The cellular components, molecular functions, and biological processes associated with the consistent eccDNAs. **N** KEGG pathway analysis of the consistent eccDNAs between NSCLC and matched normal lung tissue. **O**, **P** Volcano plot and histogram showing that there were no differentially expressed eccDNAs between NSCLC and matched normal lung tissues. EccDNA extrachromosomal circular DNA, NSCLC non-small cell lung cancer, Mb megabase.

High-throughput sequencing revealed more than 100 million clean reads in each lung tissue sample (Table S1). By mapping these clean reads to the human genome (UCSC hg19), an average of 945 eccDNAs and 12 635 eccDNA-amplified genes in normal lung tissue samples and 1 005 eccDNAs and 12 525 eccDNA-amplified genes in NSCLC tissue samples were identified (Fig. 1A). Most of these eccDNAs were detected in more than one specimen. More than 5 eccDNAs were derived from chromosomal DNA for 16 genes (Table S2). These results indicated that eccDNAs were common in NSCLC tissues.

The genomic distribution analysis revealed that eccDNAs were derived from all 23 pairs of chromosomes. A few eccDNAs derived from mitochondrial DNA were detected. The eccDNA frequency per Mb was comparable for each chromosome, except for chromosome Y, which had a much lower frequency of eccDNAs (Fig. 1B, C). There was a significant correlation between the ratio of coding genes/Mb and eccDNA/Mb in any of the chromosomes (*r* = 0.9438, *P* = 0.0047; Fig. 1D). All the eccDNAs were mapped to different classes of genomic regions. The eccDNAs mainly originated from intergenic regions, exons, and introns. However, eccDNAs were rarely located 2 kb upstream or downstream of genes (Fig. 1E, F).

Previous studies have demonstrated that the length distribution of eccDNAs is different between lung cancer tissues and normal lung tissues [10, 30]. Thus, the length distribution of eccDNAs in NSCLC tissues and matched normal tissues was examined. The overall length distribution of the eccDNAs ranged from 0.01 kb to 1000 kb with distinctive peaks at 0.7 kb (Fig. 1G). The length distribution in NSCLC and normal lung tissues showed similar features, including the location of the peak and the span of the length of eccDNAs.

The Venn diagram showed 80 eccDNAs and 618 associated genes common to NSCLC and normal lung tissues (Fig. 1H, J). The chromosome distribution of the eccDNAs was similar between the two groups (Fig. 1I).

Bioinformatic analyses were performed to examine the functions of the genes associated with eccDNAs in NSCLC and matched lung tissues. GO analysis revealed that the genes associated with eccDNAs were enriched in the mitochondrial respiratory chain, ATP synthesis and NADH dehydrogenase complex (Fig. 1K–M). KEGG pathway analysis revealed that the genes were mainly involved in cancer-related pathways, such as the oxidative phosphorylation pathway, and the primary immunodeficiency pathway (Fig. 1N).

Next, the differential expression of eccDNAs between NSCLC and matched normal lung tissues was assessed. The edgeR (v0.6.9) software was used to perform normalization and differentially expressed eccDNA filtering by the screening criteria (*P* value < 0.05 and |LogFC| > 2). However, the filtering results revealed no differentially expressed eccDNAs between NSCLC tissues and matched normal lung tissues (Fig. 1O, P).

### Genome-wide detection and analysis of eccDNAs in NSCLC cells and normal lung cells

The differences in eccDNAs between NSCLC cells (H1299, H460 and A549) and normal lung cells (MRC-5) with a relatively simple genetic background were analyzed by the same method. We also developed a process for screening the differentially expressed eccDNAs (Fig. S1A).

**Fig. 2:**
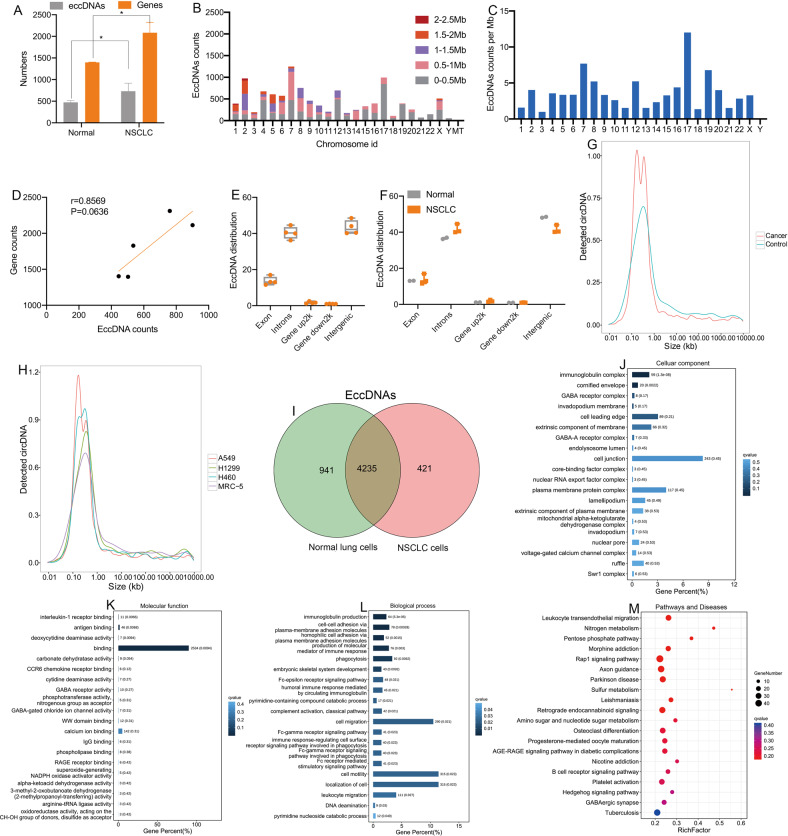
Genome-wide detection and analysis of eccDNA distribution by high-throughput sequencing in NSCLC cells and normal cells. **A** The number of eccDNA types and involved genes from normal and NSCLC cells. **B** The distribution of eccDNAs in the 23 pairs of chromosomes. **C** The eccDNA frequency per Mb in each chromosome. **D** There was no significant correlation between the number of coding genes and eccDNAs in each chromosome (*P* = 0.0636). **E**, **F** The distribution of eccDNAs in different classes of genomic regions. **G**, **H** The length distribution of eccDNA ranged from 0.01 kb to 1000 kb. **I** A Venn diagram showing the eccDNAs detected in NSCLC and normal lung cells. **J**–**L** The cellular components, molecular functions, and biological processes associated with the eccDNAs. **M** KEGG pathway analysis of the eccDNAs between NSCLC and normal lung cells. EccDNA extrachromosomal circular DNA, NSCLC non-small cell lung cancer, Mb megabase.

The results showed that the number of identified eccDNAs in NSCLC cells was larger than that in normal lung cells (Fig. 2A) (Table S3). More than 5 eccDNAs were derived from chromosomal DNA for 8 genes (Table S4). These results indicated that the presence of eccDNAs was a common event in NSCLC cells. The karyotyping results also showed the presence of circular DNA elements (Fig. S1B, C).

The genomic distribution of the eccDNAs in the cell lines was similar to that in lung tissues (Fig. 2B). Gene-rich chromosome 17 contributed more eccDNAs per megabase than other chromosomes (Fig. 2C), indicating that gene-rich chromosomes contribute more eccDNAs per megabase, consistent with a previous study [7]. There was no correlation between the ratio of coding genes/Mb and eccDNA/Mb in any of the chromosomes (*r* = 0.8569, *P* = 0.0636; Fig. 2D). The genomic regions and the length distribution of eccDNAs were also similar to the results of lung tissues (Fig. 2E–H).

The Venn diagram showed that 4 235 eccDNAs were detected in both samples (Fig. 2I). GO analysis revealed that the genes were enriched in mitochondrial function, binding and immune response (Fig. 2J–L). KEGG pathway analysis revealed that the genes associated with eccDNAs were mainly involved in leukocyte transendothelial migration and the nitrogen metabolism pathway (Fig. 2M). These results indicated that the chromosome distribution, length distribution, and genomic annotation of the eccDNAs were comparable between the NSCLC and normal groups.

### Identification of differentially expressed eccDNAs and enrichment analysis of the eccDNAs encoding amplified genes between NSCLC and normal lung cells

**Fig. 3:**
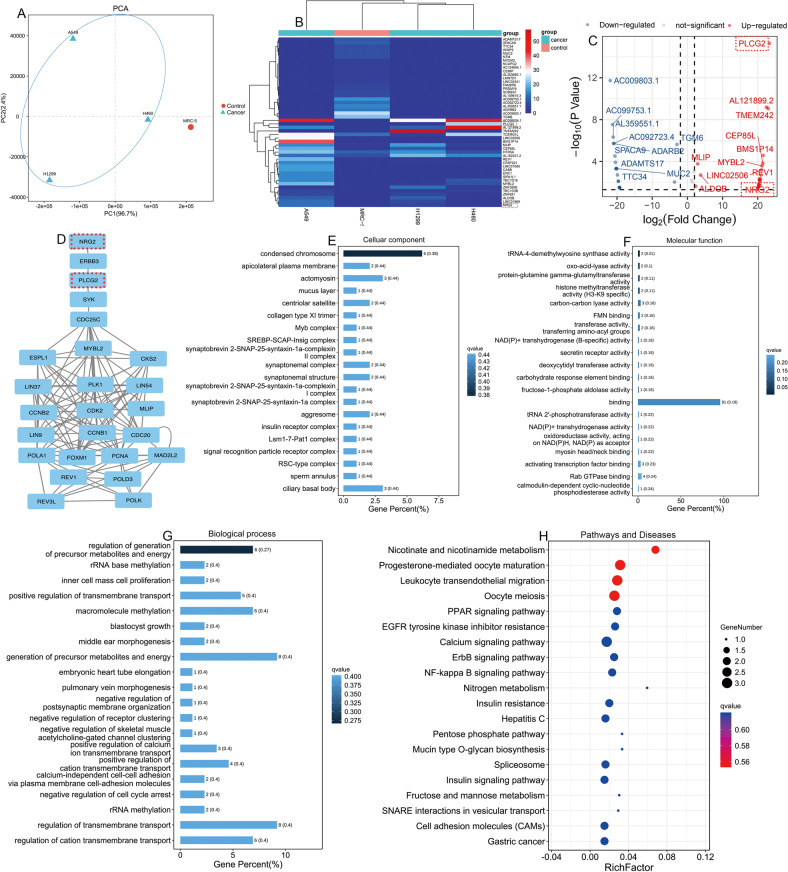
Identification of the differentially expressed eccDNAs and genes between NSCLC cells and normal cells. **A** PCA of NSCLC cells and normal cells according to differences in eccDNA-related genes. **B**, **C** Cluster and scatter plots showing the differentially expressed eccDNA-related genes between NSCLC cells and normal cells. **D** PPI analysis of the major differentially expressed eccDNA-derived genes from STRING (https://string-db.org/). **E**–**G** The cellular components, molecular functions, and biological processes associated with the differentiated eccDNA-related genes. **H** KEGG pathway analysis of the eccDNA-related genes between NSCLC and normal lung cells. EccDNA extrachromosomal circular DNA, NSCLC non-small cell lung cancer.

Principal component analysis (PCA) between NSCLC cells and normal lung cells showed that NSCLC cells could be distinguished from normal lung cells based on eccDNAs (Fig. 3A). According to the screening criteria (*P* value < 0.05 and |LogFC| > 2), differentially expressed eccDNAs between NSCLC and normal lung cells were compared. A total of 20 eccDNAs (the top 10 upregulated eccDNAs and the top 10 downregulated eccDNAs) were defined as candidate differentially expressed eccDNAs according to the fold change (Table S5) (Fig. 3B, C). Moreover, protein–protein interaction network (PPI) analysis indicated that NRG2 and PLCG2 were upstream of the other three eccDNA-related genes from STRING (https://string-db.org/) (Fig. 3D). GO analysis revealed that differentially expressed eccDNAs were enriched in condensed chromosomes, binding and generation of precursor metabolites and energy (Fig. 3E–G). The KEGG results showed that differentially expressed eccDNAs played an important regulatory role in the nicotinate and nicotinamide metabolism pathways (Fig. 3H). Therefore, these candidate upregulated eccDNAs may be potential biomarkers to distinguish NSCLC cells from normal lung cells and may participate in the initiation and progression of NSCLC.

### PLCG2 promoted the proliferation, migration, and invasion of H1299 cells

**Fig. 4:**
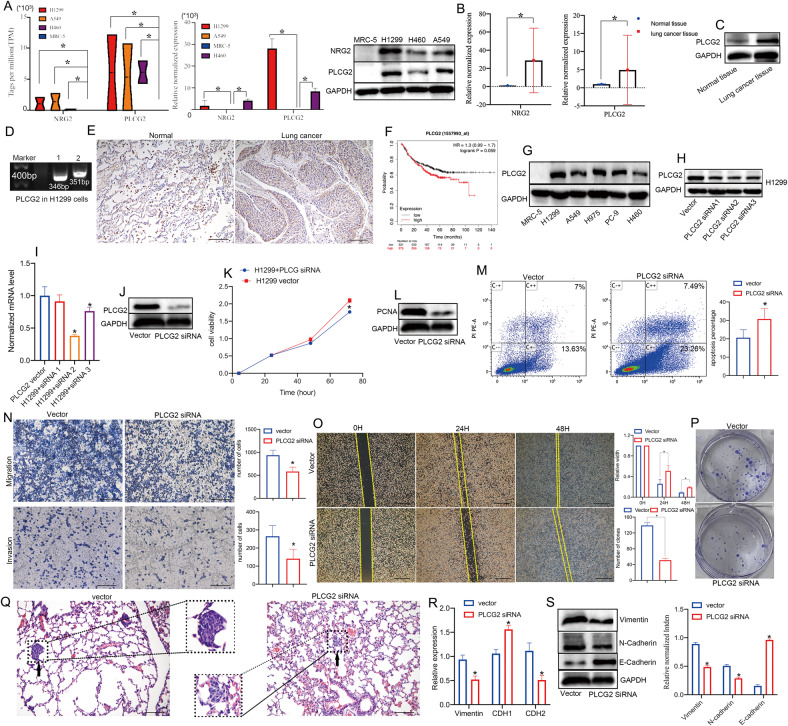
The upregulated eccDNA-related gene PLCG2 promoted the proliferation, migration, and invasion of H1299 cells. **A** The abundance (the tags per million) and the expression of NRG2 and PLCG2 in NSCLC cells and MRC-5 cells detected by whole genome resequencing, qPCR and western blotting. **B** The mRNA expression of NRG2 and PLCG2 in lung cancer tissues and normal tissues by qPCR. **C** The protein level of PLCG2 in lung cancer tissues and normal tissues by western blotting. *P < 0.05. **D** The product of circle PLCG2 in H1299 cells by PCR. **E** IHC staining showing the level of PLCG2 in lung cancer tissues and normal tissues. **F** The relationship between PLCG2 and the recurrence-free survival of NSCLC patients in the TCGA database (Kaplan‒Meier Plotter, https://kmplot.com/analysis/index.php?p=service&start=1). **G** Western blotting for PLCG2 levels of NSCLC cells and normal cells. **H**–**J** The knockdown efficiency of PLCG2 in H1299 after PLCG2 siRNA transfection by qPCR and western blotting. **K**–**M** The proliferation ability, apoptosis rate and expression of proliferating cell nuclear antigen (PCNA) in H1299 after PLCG2 siRNA transfection. **N**–**Q** The migration and invasion of H1299 cells after PLCG2 siRNA transfection by transwell assay, wound healing assay, clonogenic assay and in vivo tail injection data. **R**, **S** The levels of EMT-related biomarkers in H1299 cells after PLCG2 siRNA transfection by qPCR and western blotting. *P < 0.05.

To further explore the role of upregulated eccDNAs in NSCLC cells, based on the results of PPI analysis, we selected the upregulated eccDNA-encoding genes PLCG2 and NRG2 as our research focus. We first detected the expression levels of PLCG2 in NSCLC and normal lung cells. Compared with normal lung cells, PLCG2 was significantly upregulated in NSCLC cells, as shown by high-throughput sequencing, qPCR and western blotting (Fig. 4A). In addition, the expression of NRG2 and PLCG2 was upregulated in NSCLC tissues compared with matched normal tissues, as shown by qPCR (Fig. 4B) and western blotting (Fig. 4C). A previous study showed that PLCG2 had high expression in small cell lung cancer (SCLC) and was associated with increased stem-like and pro-metastatic potential and reduced overall survival in patients [26, 31], so we suspected that PLCG2 plays an oncogenic role in NSCLC. Therefore, we chose PLCG2 for further study. First, PCR confirmed the presence and expression of these candidate eccDNAs, which was in accordance with the results from high-throughput sequencing (Fig. 4D). The results of IHC staining also indicated that PLCG2 expression was higher in NSCLC tissues (Fig. 4E) and its level was related to the prognosis of NSCLC patients in the TCGA cohort (Fig. 4F). In addition, the expression analysis from the CPTAC samples in UALCAN online databases [32, 33] also showed a higher expression of PLCG2 in lung adenocarcinoma (LUAD) (Fig. S2A, B).

PLCG2 has been implicated in the regulation of cell proliferation, transformation, and tumor growth and reported as an important oncogene in various cancers [34, 35]. However, its mechanism in the metastasis of NSCLC has not been reported. To further explore the role of PLCG2 in NSCLC cells, we knocked down the expression of PLCG2 in H1299 cells with three individual siRNAs against PLCG2. H1299 cells had the highest expression of PLCG2 among the five NSCLC cell lines (H1299, A549, H1975, H460, and PC9) as shown by western blot (Fig. 4G). Then, we selected si2-PLCG2 for subsequent experiments since it showed the highest inhibition efficiency (Fig. 4H–J). The CCK-8 assay showed that the proliferation of the PLCG2 siRNA group was markedly inhibited (Fig. 4K), and the level of proliferating cell nuclear antigen (PCNA) was reduced in the PLCG2 siRNA group (Fig. 4L), which is a tumor cell proliferation factor related to the proliferation ability of cells. The FACS results indicated that the apoptosis rate of the PLCG2 siRNA group was higher than that of the control group (the vector group) (Fig. 4M), but the cell cycle distribution did not change (Fig. S2C). The transwell assay, wound healing assay, clonogenic assay and in vivo data indicated that knockdown of PLCG2 inhibited the migration and invasion of H1299 cells (Fig. 4N–Q). Epithelial-mesenchymal transformation (EMT) is considered to regulate multiple tumor processes, especially metastasis [36]. The levels of EMT biomarkers were detected by qPCR (CDH1, CDH2 and vimentin) and western blotting (E-cadherin, N-cadherin and vimentin). The level of E-cadherin was higher in the PLCG2 siRNA group, while the levels of vimentin and N-cadherin were lower. The changes in EMT biomarkers suggested that EMT was inhibited in the PLCG2 siRNA group (Fig. 4R, S). These results revealed that PLCG2 could exist in eccDNA form and act as an oncogene, promoting the proliferation and metastasis of NSCLC cells.

### PLCG2 enhanced mitochondrial function via the electron transport chain to promote metastasis in NSCLC

Further study was performed to explore the mechanism by which PLCG2 acts as an oncogene in H1299 cells. The results of GO and KEGG enrichment analyses showed that the differentially expressed eccDNAs were mainly related to metabolism. Metabolic reprogramming has been characterized as one of the ten most important hallmarks of malignant tumors [37]. Therefore, we hypothesized that PLCG2 could induce metabolic reprogramming to promote metastasis in NSCLC.

**Fig. 5:**
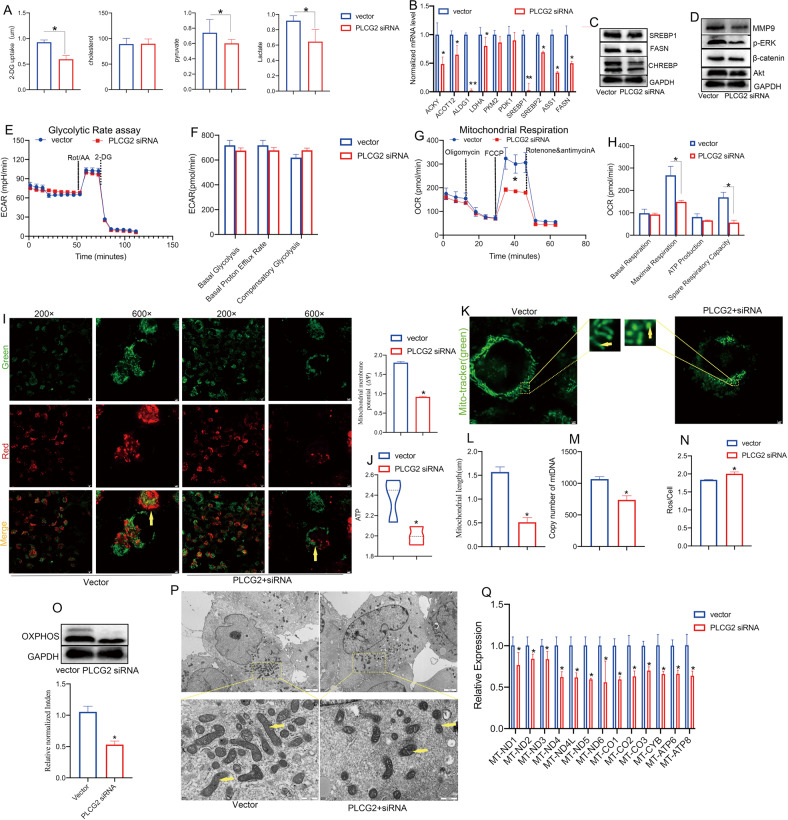
PLCG2 knockdown induced mitochondrial dysfunction by regulating the ETC. **A** Glucose uptake, cholesterol, pyruvate and lactate production in H1299 cells after PLCG2 siRNA transfection. **B**, **C** The expression of metabolism-related genes in H1299 cells after PLCG2 siRNA transfection by qPCR and western blotting. **D** The levels of the metabolic reprogramming-related β-catenin/Akt signaling pathway components in H1299 cells after PLCG2 siRNA transfection, as shown by western blotting. **E**–**H** The glycolytic rate and mitochondrial respiration of H1299 cells after PLCG2 siRNA transfection, as determined by Seahorse XF. **I** The mitochondrial membrane potential (MMP, ΔΨ) of H1299 cells after PLCG2 siRNA transfection by JC-1 staining. Scale bar = 600×. **J** Mitochondrial ATP synthesis in the PLCG2 siRNA group by the ATP detection kit. **K**, **L** The length of mitochondria in the PLCG2 siRNA group by MitoTracker Green staining and ImageJ software. **M** The number of mitochondria in the PLCG2 siRNA group as determined by mtDNA copy number count. **N** The production of reactive oxygen species (ROS) in the PLCG2 siRNA group. **O** The OXPHOS components in the PLCG2 siRNA group by western blotting. **P** Morphological changes in mitochondria in the PLCG2 siRNA group by TEM. **Q** The expression of ETC-related genes in the PLCG2 siRNA group by qPCR. Representative results from at least three independent experiments are shown. Data are represented as the mean ± SD of at least three experiments and statistically analyzed by Student’s t test or one-way ANOVA. *P < 0.05.

We first analyzed the Warburg effect by measuring glucose uptake and pyruvate and lactate production. The results showed that PLCG2 siRNA inhibited the Warburg effect (aerobic glycolysis) but did not change the cholesterol concentration (Fig. 5A). The expression of intermediate metabolites and kinase-related metabolites were decreased in the PLCG2 siRNA group according to qPCR (ACLY, ACOT12, ALDG1, LDHA, PKM2, PDK1, SREBP1, SREBP2, ASS1, and FASN) and western blotting (SREBP1, FASN, and CHREBP1) (Fig. 5B, C). The β-catenin/Akt signaling pathway, which is related to metabolic reprogramming [38], was also suppressed in the PLCG2 siRNA group (Fig. 5D). PLCG2 also promoted mitochondrial respiration, whereas the glycolytic rate did not markedly increase (Fig. 5E-H). These results showed that PLCG2 may promote the metastasis of NSCLC cells by enhancing mitochondrial respiration.

**Fig. 6:**
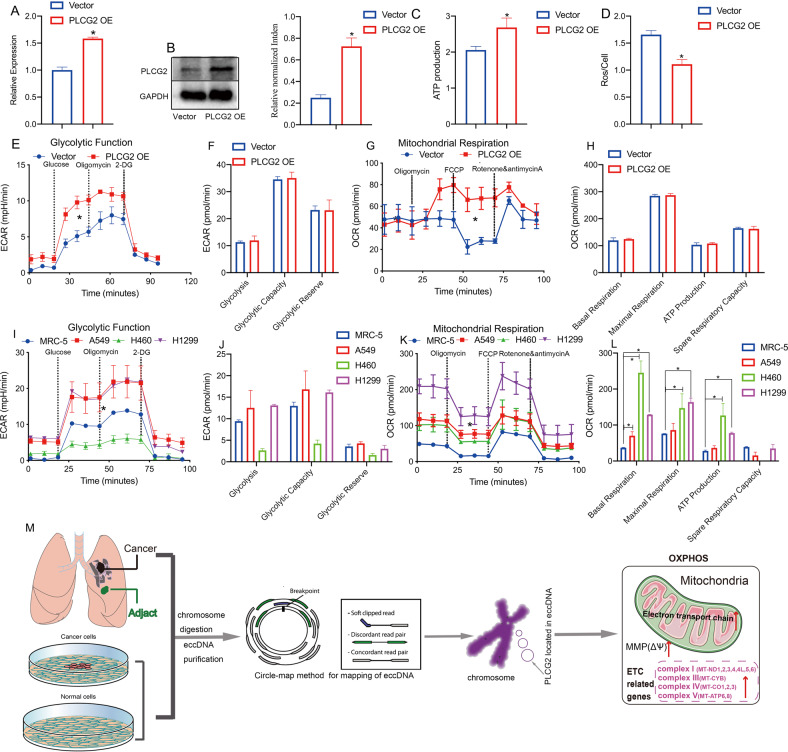
High level of PLCG2 in NSCLC cells contributed to the higher mitochondrial respiration. **A**, **B** The overexpression of PLCG2 in H460 cells after transfection, confirmed by qPCR and western blotting. **C** Mitochondrial ATP synthesis in the PLCG2 OE group by the ATP detection kit. **D** The production of reactive oxygen species (ROS) in the PLCG2 OE group. **E**–**H** The glycolytic function and mitochondrial respiration of H460 cells in PLCG2 OE group, as determined by a Seahorse XF Analyzer. **I**–**L** The glycolytic function and mitochondrial respiration of H1299, A549, H460 and MRC-5, with different levels of PLCG2, as determined by a Seahorse XF Analyzer. **M** Schematic representation of the role and mechanism of eccDNAs in the metastasis of NSCLC cells.

We next explored the mechanism by which PLCG2 enhanced mitochondrial respiration in H1299 cells. JC-1 staining was used to detect mitochondrial membrane potential (MMP, ΔΨ), and MitoTracker staining and transmission electron microscopy (TEM) were used to observe mitochondrial morphology. Both MMP and ATP were decreased in the PLCG2 siRNA group (Fig. 5I, J). MitoTracker staining (green) showed that mitochondrial length was decreased (Fig. 5K, L) and the number of mitochondria in the PLCG2 siRNA group was reduced, as determined by mtDNA copy number count (Fig. 5M). Moreover, the level of ROS was elevated, while the expression of OXPHOS components was decreased in the PLCG2 siRNA group (Fig. 5N, O). The TEM results revealed mitochondrial pyknosis, darker staining, a coarser mitochondrial crest, and larger lacuna in the PLCG2 siRNA group (Fig. 5P), which also confirmed that PLCG2 was associated with mitochondrial function. The integrity of the mitochondrial respiratory chain, also known as the electron transport chain (ETC), depends on a number of proteins encoded by nuclear and mitochondrial genomes. Mutations in such factors can result in one or more respiratory chain defects, and some mutations can alter the morphology of the mitochondrial network or induce the accumulation of intermediary metabolites [39]; thus, we assessed the 13 respiratory chain proteins encoded by mtDNA (mt-ND1, mt-ND2, mt-ND3, mt-ND4, mt-ND4L, mt-ND5, mt-ND6, mt-CO1, mt-CO2, mt-CO3, mt-CYB, mt-ATP6, and mt-ATP8) by RT‒qPCR, with Cytochrome C Oxidase Subunit IV Isoform 1 (COX4I1) as the mitochondrial housekeeping gene. The results showed that PLCG2 siRNA inhibited the ETC, which suggested that PLCG2 may enhance mitochondrial function via the ETC (Fig. 5Q). In addition, the changes of mitochondrial respiration in the PLCG2-overexpression (OE) H460 cells and the four cell lines with different levels of PLCG2 (H1299, A549, H460 and MRC-5) indicated that high level of PLCG2 in NSCLC cells contributes to the higher mitochondrial respiration, which suggested that PLCG2 can enhance mitochondrial function (Fig. 6A–H, I–L).

In summary, the decreased mitochondrial membrane potential and reduced mitochondrial ATP synthesis indicated impaired mitochondrial function caused by ETC inhibition. Our results suggested that PLCG2 can exist in eccDNA form and act as an oncogene that enhances mitochondrial respiration via ETC to promote the metastasis of NSCLC cells, which further illustrated that genes in the form of eccDNA may play a role in promoting cancer.

## Discussion

EccDNAs have long been known to exist in normal tissues and cancer tissues [15]. In this study, high-throughput sequencing demonstrated the presence of eccDNAs in the matched NSCLC and normal tissues and in cell lines. Consistent with previous studies, we detected abundant eccDNAs with sizes from 0.01 kb to 1000 kb [15, 39, 40]. The eccDNAs were mapped to any region of the human genome [27]. We also found that chromosome 17, a gene-rich chromosome, was the most common site from which eccDNA was derived in NSCLC cells, and the least common site was the Y sex chromosome, similar to previous study [7]. The results of mapping the eccDNAs to various genomic regions demonstrated that they originated mainly from intergenic regions, exons, introns, and less frequently from 2 kb upstream or downstream of genes. However, no significantly upregulated or downregulated candidate eccDNAs were identified in NSCLC tissue samples. This can be explained by the fact that variations in eccDNA abundance in patients with cancer may be difficult to detect because rolling circle amplification with random primers would result in a massive loss of information related to the level of a specific eccDNA, as previously reported [15]. To further compare the eccDNA differences between NSCLC and normal groups, we used the same method to analyze NSCLC cell lines and normal lung cell lines with relatively simple genetic backgrounds.

We thoroughly analyzed the differences in eccDNA expression profiles between NSCLC cells and normal lung cells. Although the size and type of eccDNAs did not significantly differ between NSCLC cells and normal lung cells, the number of eccDNAs was significantly different between these cell types. Then, we selected several upregulated genes expressed as eccDNAs for verification. On this basis, we combined bioinformatics analysis methods to determine and verify the potential mechanism of PLCG2 eccDNA in NSCLC cells. By analyzing the differences in gene expression and eccDNA expression between NSCLC cells and normal lung cells, we found that the expression levels of 2 307 eccDNAs and 1 509 genes were significantly different between the two groups. The expression of the upregulated candidate eccDNA-encoding PLCG2 was successfully validated using routine PCR. However, the function and mechanism of PLCG2 in the progression of NSCLC were still unknown. In our study, we hypothesized that PLCG2, which exists in eccDNA, promotes metastasis by enhancing mitochondrial respiration via the ETC and plays important roles in the progression of NSCLC. Our study provides a new direction on the progression mechanism and clinical therapies of NSCLC.

There were several limitations to our study. First, we aimed to select the differentially expressed eccDNAs in NSCLC by high-throughput sequencing to explore the role of the genes whose sequences they harbor or related genes in NSCLC and their potential as NSCLC biomarkers; we focused only on protein-coding genes, while the characteristics of eccDNA, such as its structure and formation, were not deeply explored. Second, we screened only the upregulated eccDNAs containing protein-coding genes as candidates and excluded those containing only noncoding genes, such as circRNAs, lncRNAs and microRNAs. We did not clarify whether the screened highly expressed eccDNA was derived from the full-length sequence or part of PLCG2. Finally, our screened upregulated eccDNAs were based on results in NSCLC cell lines, not NSCLC tissues. The high-throughput results were validated, but further research with more clinical samples is needed.

## Conclusion

To the best of our knowledge, this study was the first to combine NSCLC tissue and cell line samples to analyze eccDNA characteristics, including the size distribution, chromosome location and expression level of eccDNAs, by high-throughput sequencing. Based on the analysis of eccDNA sequencing data, we determined that PLCG2 can exist in eccDNA form and is highly expressed in NSCLC. Moreover, our results indicated that PLCG2, which was selected by eccDNA sequencing, acts as an oncogene that promotes the metastasis of NSCLC by enhancing mitochondrial function (Fig. 6M).

## Supplementary information

supplemental material

original western blots

AJ Checklist

## Data Availability

The datasets used and analyzed during the current study are available from the corresponding author on reasonable request.
