# Supplementary material for: PLCG2 can exist in eccDNA and contribute to the metastasis of non-small cell lung cancer by regulating mitochondrial respiration
Source: Cell Death Dis. 2023 Apr 8;14(4):257. doi: 10.1038/s41419-023-05755-7 (PMC10082821; doi:10.1038/s41419-023-05755-7)
Supplement: Supplementary file 1 — supplemental material [file 41419_2023_5755_MOESM1_ESM.docx]

**Supplementary materials**

**
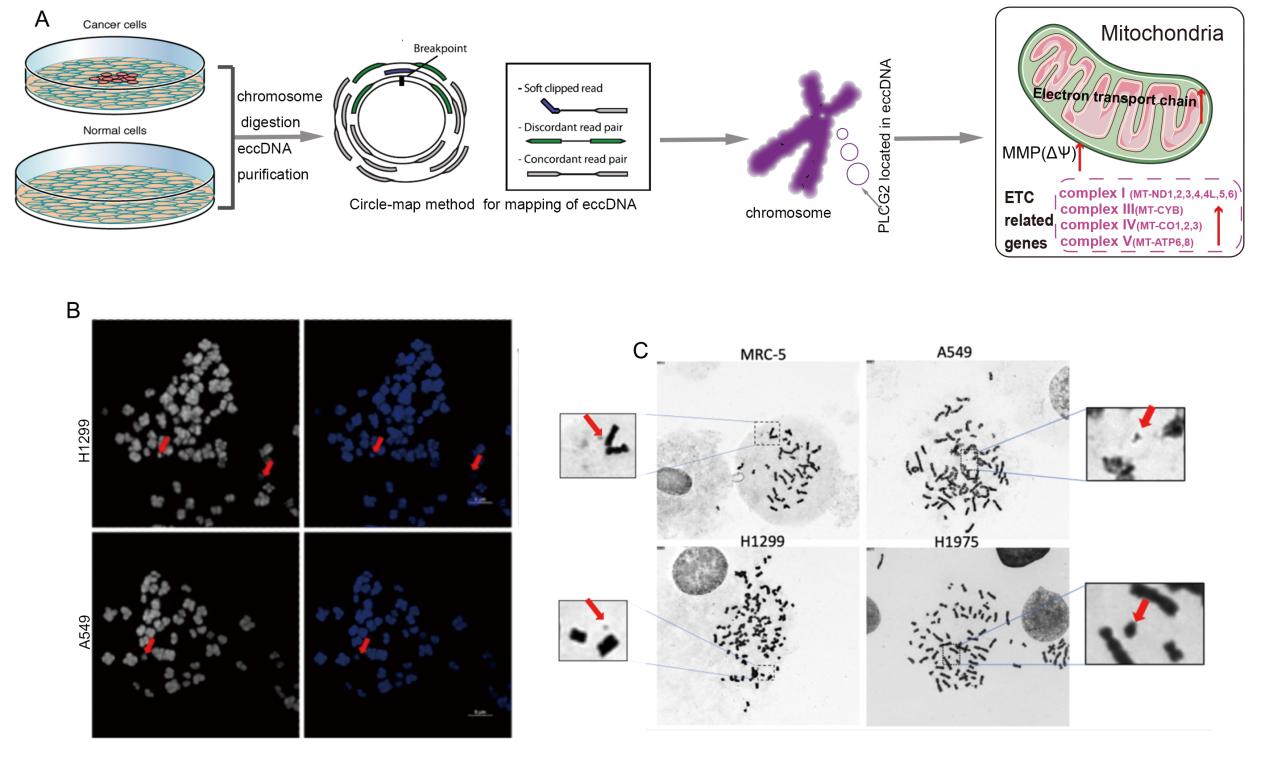
**

**Figure S1** EccDNAs were present in lung normal and NSCLC cells. (A) Workflow to explore the mechanism of oncogenes encoded by eccDNA in NSCLC.(B) Confocal micrograph for eccDNA in DAPI-stained cells in metaphase, Scal bar=600×, which indicated by the red arrows. (C) Karyotyping analysis was used to detect eccDNA in all cell lines, which indicated by the red arrows.


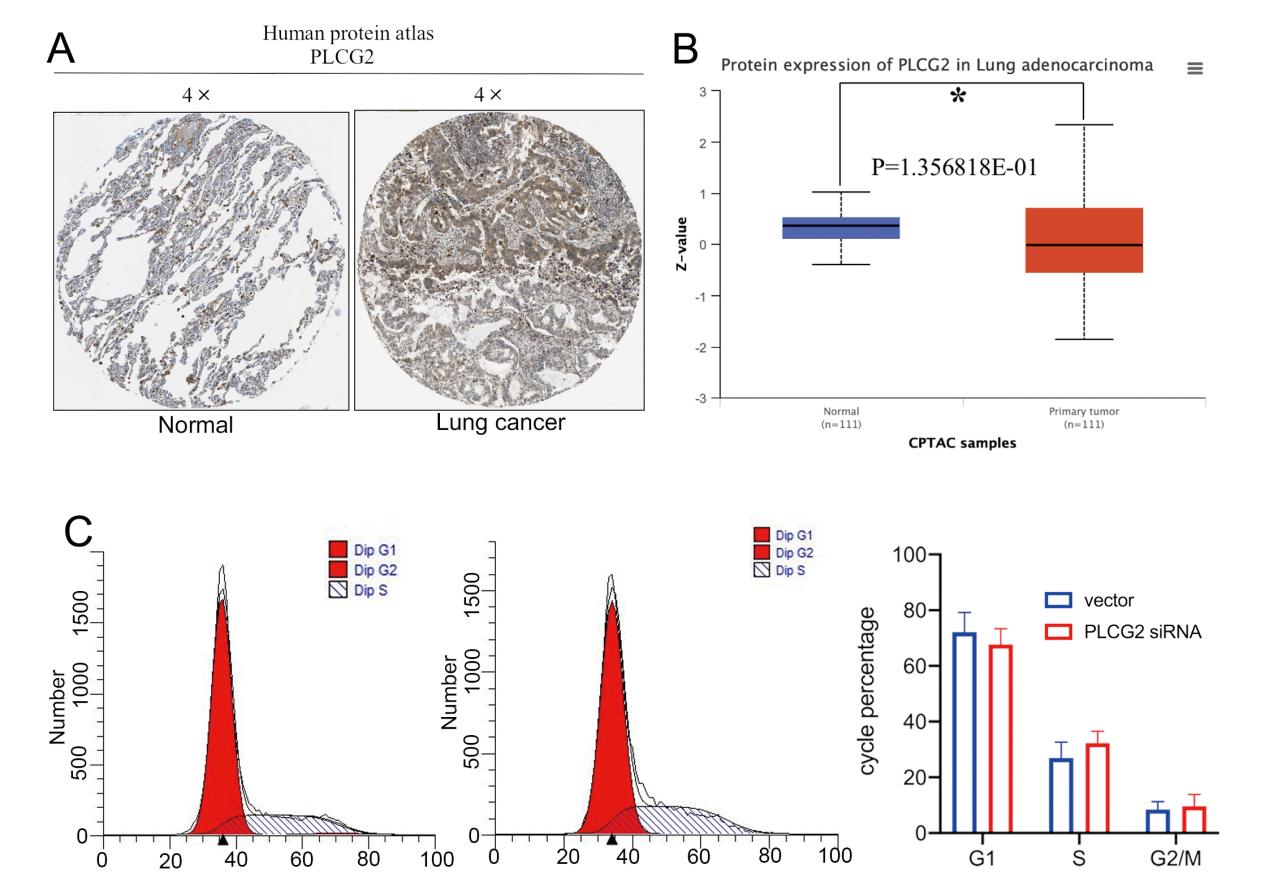


**Figure S2** The level of PLCG2 in NSCLC. (A) The protein expression of NRG2 and PLCG2 in normal lung tissue and NSCLC specimens. Images are from the Human Protein Atlas online database (http://www.proteinatlas.org). (B) Protein expression of PLCG2 in LUAD. Images were taken from the UALCAN online database (http://ualcan.path.uab.edu/index.html). (C) The cell cycle distribution of H1299 cells after PLCG2 siRNA transfection.

**Table S1** The summary of reads and eccDNA count in each tissue sample

| Reads or count | C1 | C2 | C3 | N1 | N2 | N3 |
| --- | --- | --- | --- | --- | --- | --- |
| Raw reads | 245656912 | 235765612 | 225228536 | 212569952 | 242820674 | 230825656 |
| Clean reads | 244401064 | 234501330 | 224092214 | 210880968 | 241365874 | 229286698 |
| EccDNA count | 1234 | 797 | 984 | 732 | 908 | 1184 |

Cx indicated LC, while Nx indicated matched normal epithelium. EccDNA, extrachromosomal circular DNA; LC, lung cancer.

**Table S2** The genes giving rise to more than 5 eccDNAs in tissue sample (including 5）

| Gene | Number of eccDNAs generated by the gene |
| --- | --- |
| CAMTA1 | 6 |
| CDH4 | 10 |
| CNTNAP2 | 6 |
| CSF2RA | 5 |
| CTNND2 | 5 |
| DLGAP2 | 8 |
| DYSF | 6 |
| HIVEP3 | 5 |
| KAZN | 5 |
| LYPD8 | 5 |
| PTPRN2 | 18 |
| TAFA5 | 5 |
| TMEM114 | 6 |
| TPO | 9 |
| TTC34 | 7 |
| ZFYVE28 | 6 |

EccDNA,extrachromosomal circular DNA.

**Table S3** The summary of reads and eccDNA count in each cell sample

| Reads or count | A549 | H1299 | H460 | MRC-5 |
| --- | --- | --- | --- | --- |
| Raw reads | 280471888 | 185551080 | 270124206 | 242552626 |
| Clean reads | 278969916 | 184808258 | 268666984 | 242164360 |
| EccDNA count | 901 | 535 | 760 | 503 |

EccDNA, extrachromosomal circular DNA

**Table S4** The genes giving rise to more than 5 eccDNAs in cell sample (including 5)

| Gene | Number of eccDNAs generated by the gene |
| --- | --- |
| DLGAP2 | 5 |
| GRK5 | 5 |
| HIVEP3 | 5 |
| MIR646HG | 6 |
| MTUS2 | 5 |
| PRDM16 | 5 |
| PTPRN2 | 20 |
| SLC45A2 | 5 |

EccDNA,extrachromosomal circular DNA.

**Table S5** The genes generating the most candidate eccDNAs at differential level (top 10)

| Up-regulated eccDNAs in NSCLC cells | | Down-regulated eccDNAs in NSCLC cells | |
| --- | --- | --- | --- |
| Gene | Count of eccDNAs generated by the gene | Gene | Count of eccDNAs generated by the gene |
| PLCG2 | 2 | ADARB2 | 1 |
| TMEM242 | 3 | SPACA9 | 1 |
| AL121899.2 | 1 | ADAMTS17 | 1 |
| BMS1P14 | 1 | TTC34 | 2 |
| CEP85L | 2 | WWP2 | 3 |
| MYBL2 | 3 | MUC2 | 4 |
| REV1 | 1 | NTM | 2 |
| NNT | 1 | FAM87B | 2 |
| ZNF385B | 1 | PRDM16 | 5 |
| NRG2 | 2 | LMNTD1 | 1 |

EccDNA, extrachromosomal circular DNA; NSCLC, Non-small cell Lung cancer.

**Table S6** The primers of candidate up-regulated eccDNAs in NSCLC cells

| Candidate eccDNAs | Forward primer | Reverse primer | Product size |
| --- | --- | --- | --- |
| PLCG2(chr16:81765054-81765595) | 5’-GCAAACCAGAACACACAAAAG-3’ | 5’-TGGAGCACTAGCACCACCTC-3’ | 541 |
| PLCG2(chr16:81765054-81765621) | 5’-CAAACCAGAACACACAAAAGATT-3’ | 5’-GCTGGATCTCACTCTGTCATC-3’ | 567 |

In the form of ChrX:Y-Z, X indicates chromosome id, Y indicated start size and Z indicates end site. EccDNA, extrachromosomal circular DNA; NSCLC, Non-small cell lung cancer.

**Table S7** The sequence of PLCG2 located on extrachromosomal circle DNA

| The name of located eccDNAs | The sequence |
| --- | --- |
| EccDNA00000674  (chr16:81765054-81765595) | CCTCCGGAAGAAAGCAACCATGCCGGCCAGGTGTGGAGGCTCATGCCTGTAATTCCAGCACTTTGGGAGGCCCAGGAGGGCAGATCACTTGAGGTCAGGAGTTCAAGACCAGCCTGGGCTACATGATGAAACCCTGTTTCTACATAAAATACAAAAATTAGCTGGGCGTCATGATGGGCACCTGTAATCCCAGCTACCCGGGAGGCTGAGGCAGGAGAATTGCTTGGACCCGAGAAGCAGAGGTTGCTGTGAGCTGAGGTGGTGCTAGTGCTCCAGCCTGGGTGACAGAGTGAGATGAGACACTGTCCCCCCACAAAAAAAATAAATCAATGACTGGTGTGTCATAAGAAGAGGGACATTTGGACACAGAGACATGCACAGAGGGAAGATGATATGAAGGCAACACAGCGACACACAGGGAGAGCACCATGTGACAACTGAGGCAGAGGATGGGGAGAGGCATCTGCAAACCAGAACACACAAAAGATTCCCAGCAACCACCCAAAGCTTGGAGAGAGGCATGGGACAATCTCCCGCAGAG |
| EccDNA00000675  (chr16:81765054-81765621) | GCAACCATGCCGGCCAGGTGTGGAGGCTCATGCCTGTAATTCCAGCACTTTGGGAGGCCCAGGAGGGCAGATCACTTGAGGTCAGGAGTTCAAGACCAGCCTGGGCTACATGATGAAACCCTGTTTCTACATAAAATACAAAAATTAGCTGGGCGTCATGATGGGCACCTGTAATCCCAGCTACCCGGGAGGCTGAGGCAGGAGAATTGCTTGGACCCGAGAAGCAGAGGTTGCTGTGAGCTGAGGTGGTGCTAGTGCCCTCCAGCCTGGATGACAGAGTGAGATCCAGCCTGGGTGACAGAGTGAGATGAGACACTGTCCCCCCACAAAAAAAATAAATCAATGACTGGTGTGTCATAAGAAGAGGGACATTTGGACACAGAGACATGCACAGAGGGAAGATGATATGAAGGCAACACAGCGACACACAGGGAGAGCACCATGTGACAACTGAGGCAGAGGATGGGGAGAGGCATCTGCAAACCAGAACACACAAAAGATTCCCAGCAACCACCCAAAGCTTGGAGAGAGGCATGGGACAATCTCCCGCAGAGCCTCCGGAAGAAA |

**Table S8** The primer of qPCR

| Name | Forward primer | Reverse primer |
| --- | --- | --- |
| GAPDH | 5’-ATTCCACCCAGGCAAATTC-3’ | 5’-GATGGGATTTCCATTGATGACA-3’ |
| COX4I1 | 5’-TGGCGGCAGGTGTACATTTT-3’ | 5’-AGTCTTCGCTCTTCACAACACT-3’ |
| MT-ND1 | 5’-CCTCTGATTACTCCTGCCATCA-3’ | 5’-CGGCGTATTCGATGTTGAAG-3’ |
| MT-ND2 | 5’-AGCGCTAAGCTCGCACTGAT-3’ | 5’-CTTGATGGCAGCTTCTGTGG-3’ |
| MT-ND3 | 5’-TTACGAGTGCGGCTTCGACC-3’ | 5’-TTGTAGGGCTCATGGTAGGGGT-3’ |
| MT-ND4 | 5’-CGGCGCAGTCATTCTCATAA-3’ | 5’-ATGCGACTGTGAGTGCGTTC-3’ |
| MT-ND4L | 5’-TCGCTCACACCTCATATCCTC-3’ | 5’-AAGACTAGTATGGCAATAGGCACAA-3’ |
| MT-ND5 | 5’-CCACTCTGTTCGCAGCAGTC-3’ | 5’-TGCAGGAATGCTAGGTGTGG-3’ |
| MT-ND6 | 5’-ACAGCGATGGCTATTGAGGA-3’ | 5’-CAGCACCAATCCTACCTCCA-3’ |
| MT-CYB | 5’-CATCCAACATCTCCGCATGA-3’ | 5’-GGTTGAGGCGTCTGGTGAGT-3’ |
| MT-CO1 | 5’-GTTCGCCGACCGTTGACTAT-3’ | 5’-CAGCTCGGCTCGAATAAGGA-3’ |
| MT-CO2 | 5’-TACACCGACTACGGCGGACT-3’ | 5’-AACGTCAAGGAGTCGCAGGT-3’ |
| MT-CO3 | 5’-TCCTAATGACCTCCGGCCTA-3’ | 5’-GTGTTACATCGCGCCATCAT-3’ |
| MT-ATP6 | 5’-CAGGCCACCTACTCATGCAC-3’ | 5’-GGCTTGGATTAAGGCGACAG-3’ |
| MT-ATP8 | 5’-ATACTACCGTATGGCCCACCA-3’ | 5’-GGGCTTTGGTGAGGGAGGTA-3’ |
| NRG2 | 5’-ACAGCGGAAGCAGATGCAC-3’ | 5’-GTTTCTCTCCTGATGACATGGTC-3’ |
| PLCG2 | 5’-CCTTTGTGGAGACGAAGGCT-3’ | 5’-TTGTCCCTTTGGGTAGACGC-3’ |
| ALDH1 | 5’-CCGACTTGGACAATGCT-3’ | 5’-ATCCTGGATGCGGCTAT-3’ |
| HIF-1α | 5’-TCATCCATGTGACCATGAGG-3’ | 5’-TTCTTCCTCGGCTAGTTAGGG-3’ |
| Glut1-F | 5’-CGGGCCAAGAGTGTGCTAAA-3’ | 5’-TGACGATACCGGAGCCAATG-3’ |
| LDHA | 5’-TCTTGACCTACGTGGCTTGGA-3’ | 5’-CCATACAGGCACACTGGAATCTC-3’ |
| PDK1 | 5’-ACCAGGACAGCCAATACAAG-3’ | 5’-CCTCGGTCACTCATCTTCAC-3’ |
| TNF-α | 5’-TGTTGTAGCAAACCCTCAAGC-3’ | 5’-TGAAGAGGACCTGGGAGTAGAT-3’ |
| SCID | 5’-GATGTCTATGAATGGGCTCG-3’ | 5’-GGTTTGTAGTACCTCCTCTGG-3’ |
| FASN | 5’-TCCTCGCTGCCTACTGG-3’ | 5’-CGCTGTTTACACTCCTCCC-3’ |
| ACLY | 5’-CACCGAAGACCAACATCCA-3’ | 5’-CAGCAGGTAGCAGAGCAAA-3’ |
| ACC | 5’-TAACCTTGAGACTATCTGGCTTGG-3’ | 5’-TTCGTGAGGGTAATCGGAGC-3’ |
| PKM2 | 5’-AATCACGCTGGATAACGC-3’ | 5’-AAGTCGGCACCTTTCTG-3’ |
| VIM | 5’-TTGACGCAAAGTGGAATC-3’ | 5’-AGGTCAGGCTTGGAAACA-3’ |
| CDH2 | 5’-ATCCTACTGGACGGTTCG-3’ | 5’-TTGGCTAATGGCACTTGA-3’ |
| CDH1 | 5’-CTGAGAACGAGGCTAACG-3’ | 5’-GTCCACCATCATCATTCAATAT-3’ |

**Table S9** The antibody of western blot

| Name | Company | Cat. |
| --- | --- | --- |
| GAPDH | CST | 5174S |
| PLCG2 (for tissues) | abways | CY8557 |
| PLCG2 (for cells) | Santa | Sc-5283 |
| NRG2 | Santa | sc-398594 |
| PCNA | Proteintech | 10205-2-AP |
| Vimentin | CST | 5741 |
| N-Cadherin | CST | 13116S |
| E-Cadherin | Proteintech | 20874-2-AP |
| SREBP1 | affinity | AF6283 |
| FASN | Proteintech | 10624-2-AP |
| CHREBP | Novus Bio | 1V13400-135 |
| MMP9 | Proteintech | 10375-2-AP |
| p-ERK | CST | 4695S |
| β-catenin | CST | 8480S |
| AKT | CST | 4658S |
| Total OXPHOS Rodent WB Antibody Cocktail | abcam | ab110413 |
