# Supplementary material for: PLCG2 can exist in eccDNA and contribute to the metastasis of non-small cell lung cancer by regulating mitochondrial respiration
Source: Cell Death Dis. 2023 Apr 8;14(4):257. doi: 10.1038/s41419-023-05755-7 (PMC10082821; doi:10.1038/s41419-023-05755-7)
Supplement: Supplementary file 2 — original western blots [file 41419_2023_5755_MOESM2_ESM.docx]

original western blots


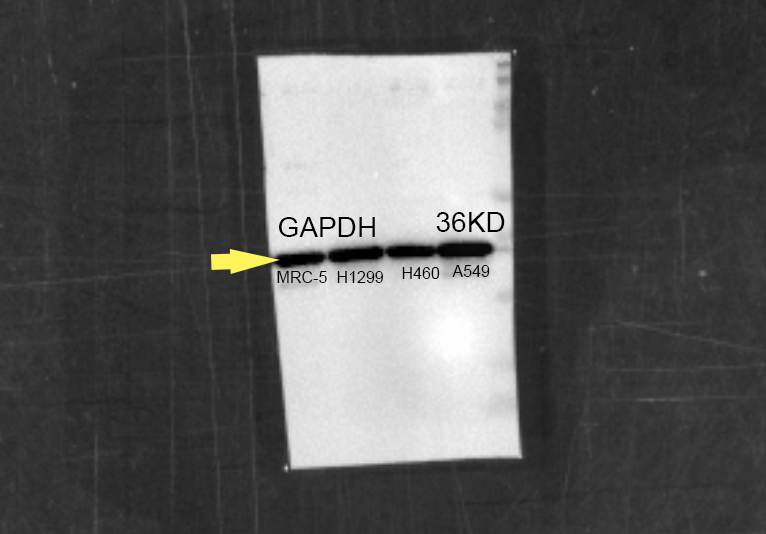


Fig4A GAPDH


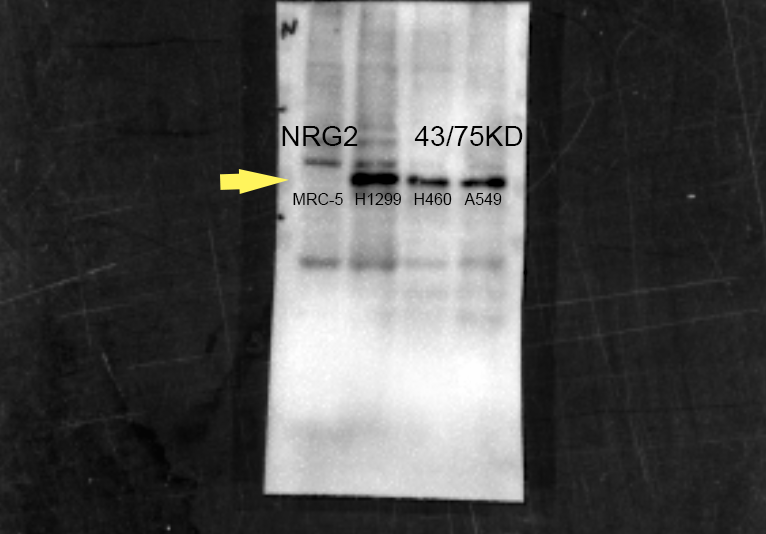


Fig4A NRG2


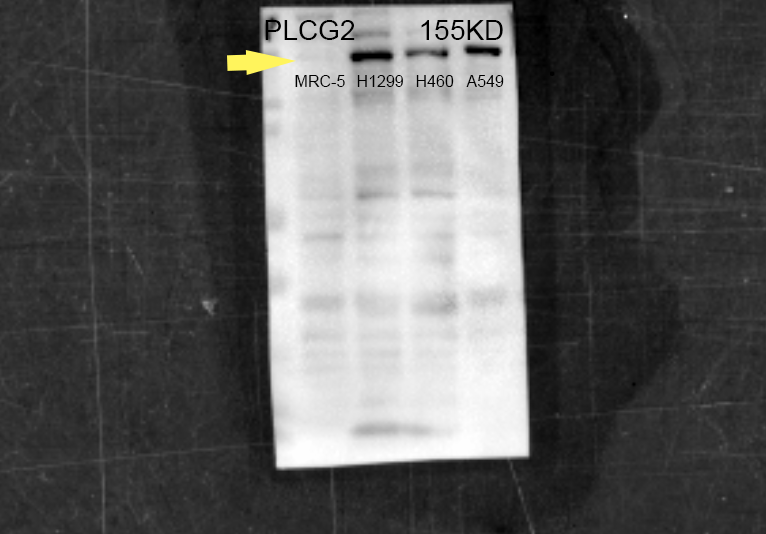


Fig4A PLCG2


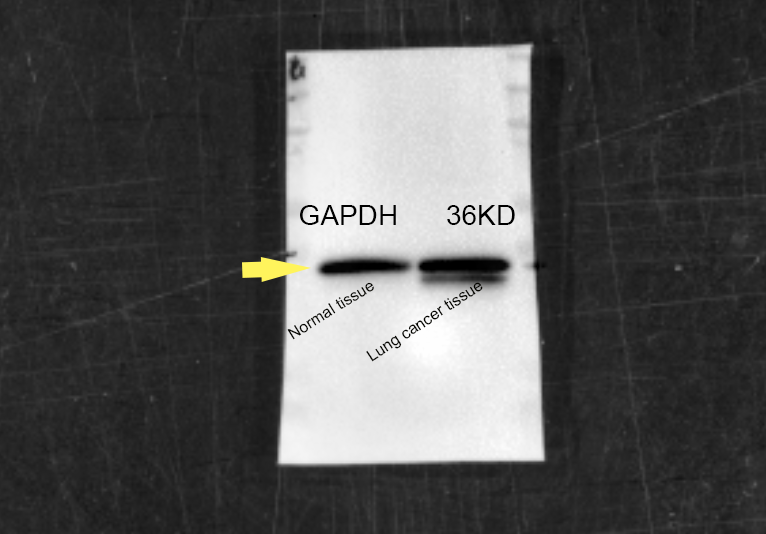


Fig4C GAPDH


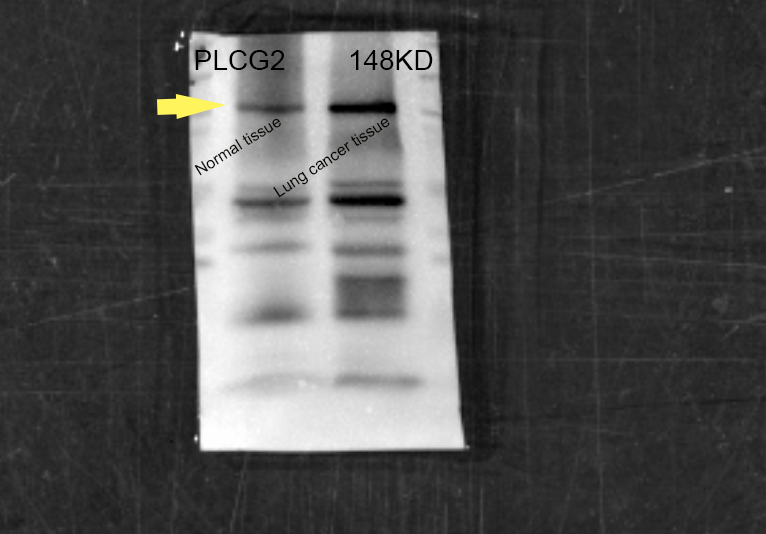


Fig4C PLCG2


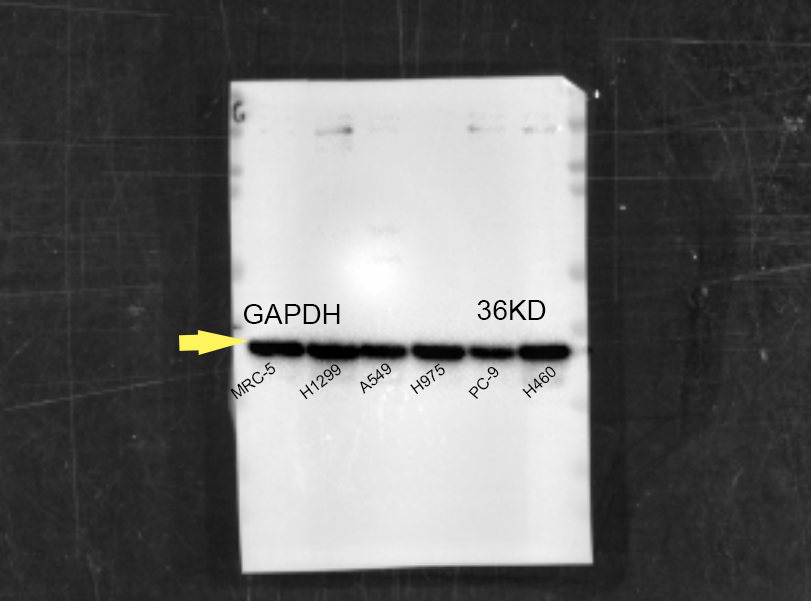


Fig4G GAPDH


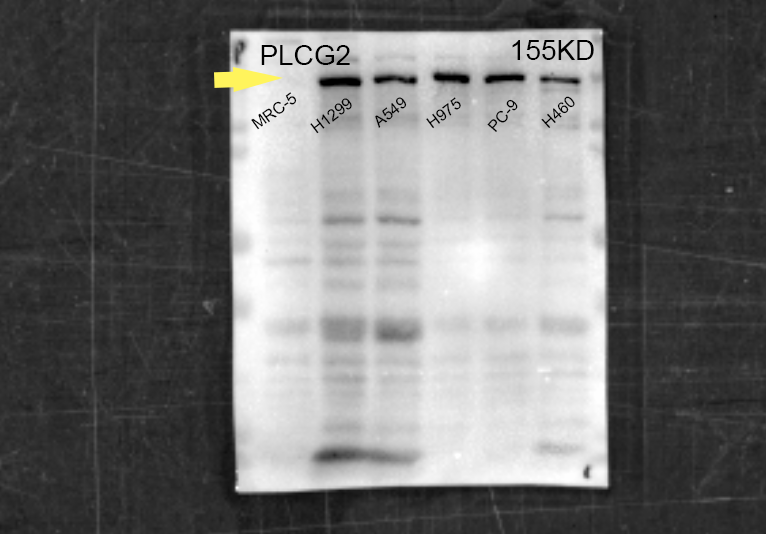


Fig4G PLCG2


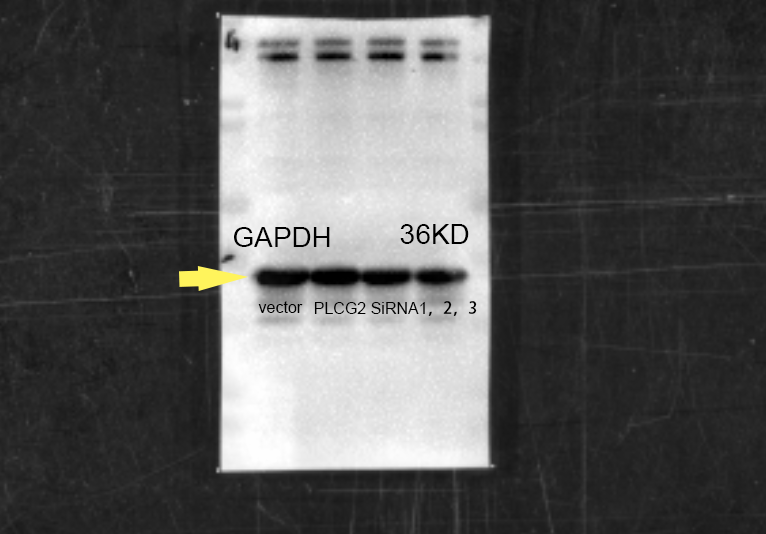


Fig4H GAPDH


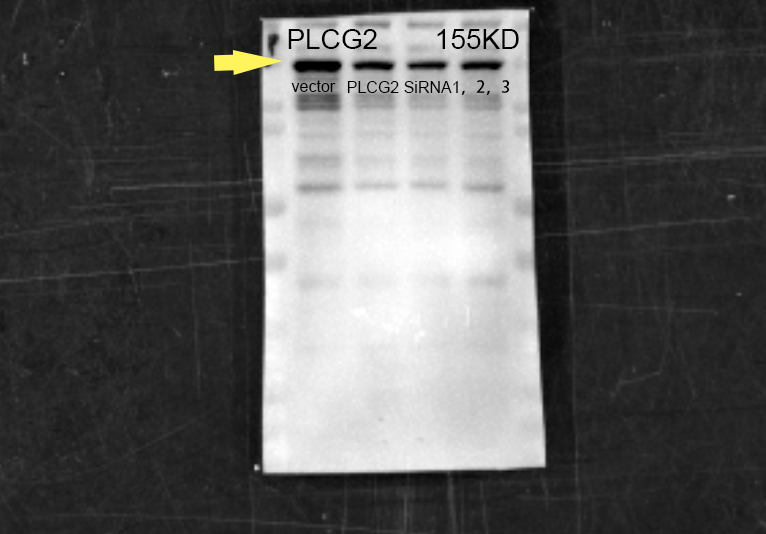


Fig4H PLCG2


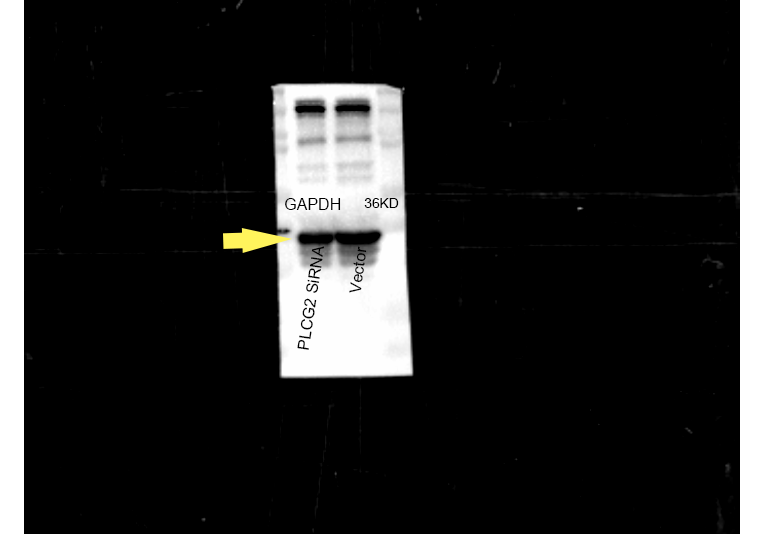


Fig4J GAPDH


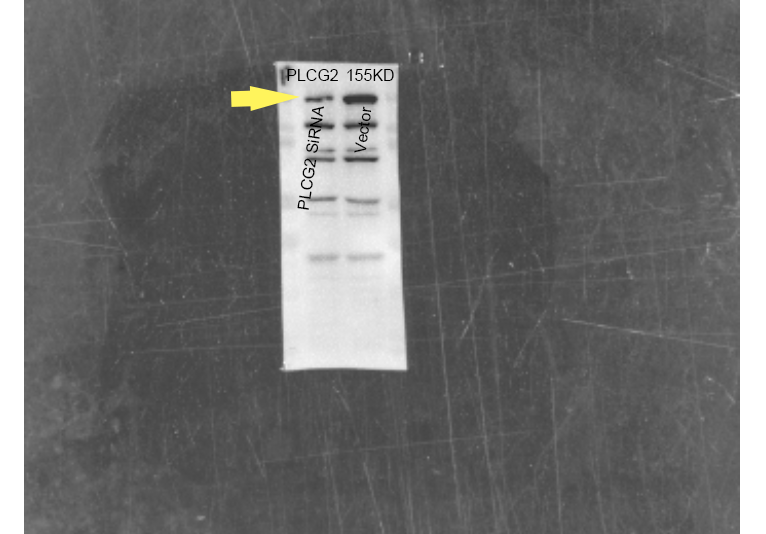


Fig4J PLCG2


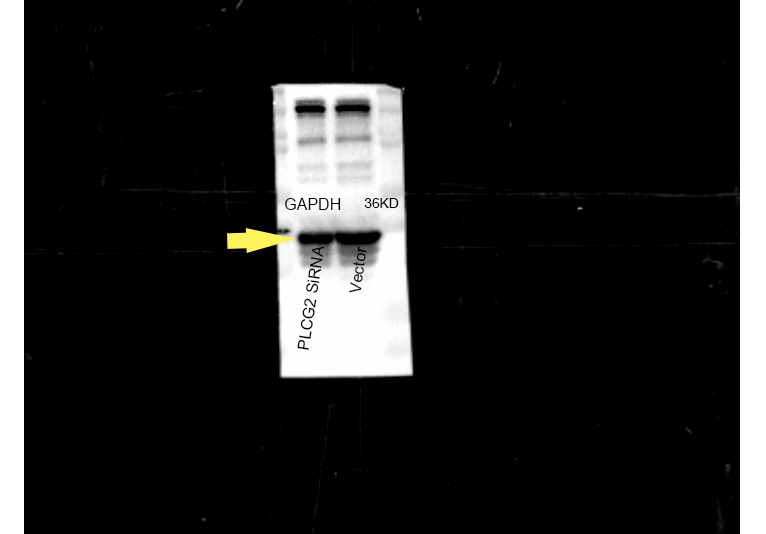


Fig4L GAPDH


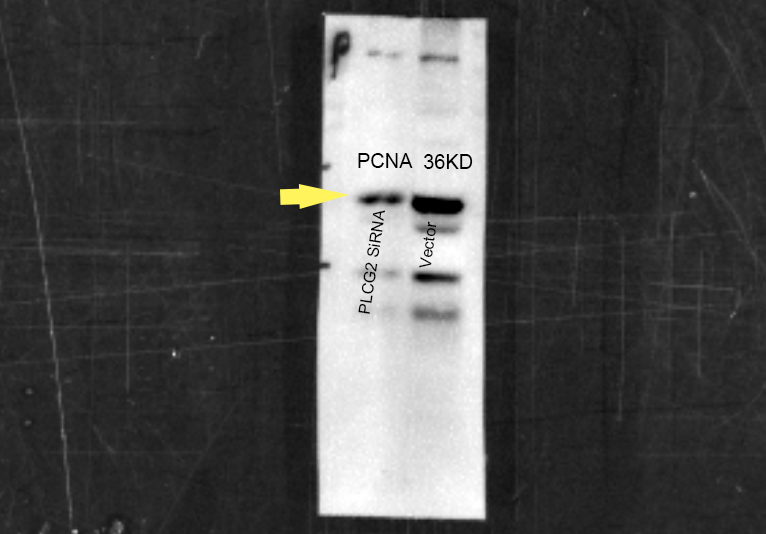


Fig4L PCNA


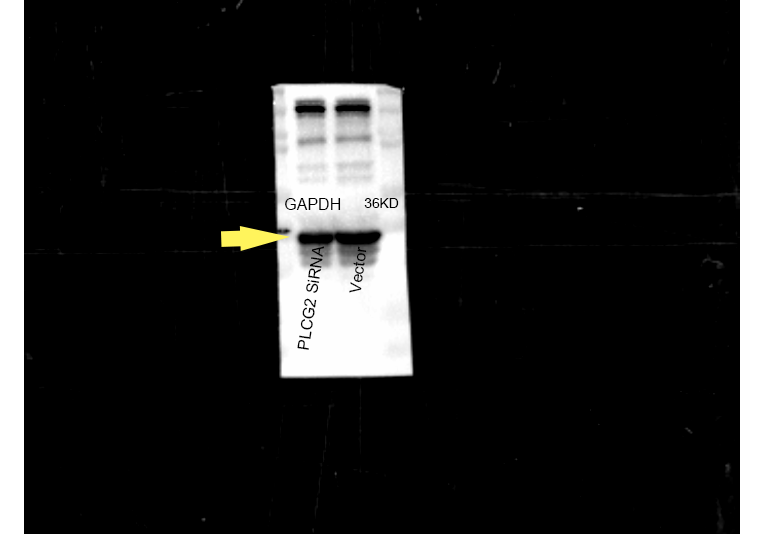


Fig4S GAPDH


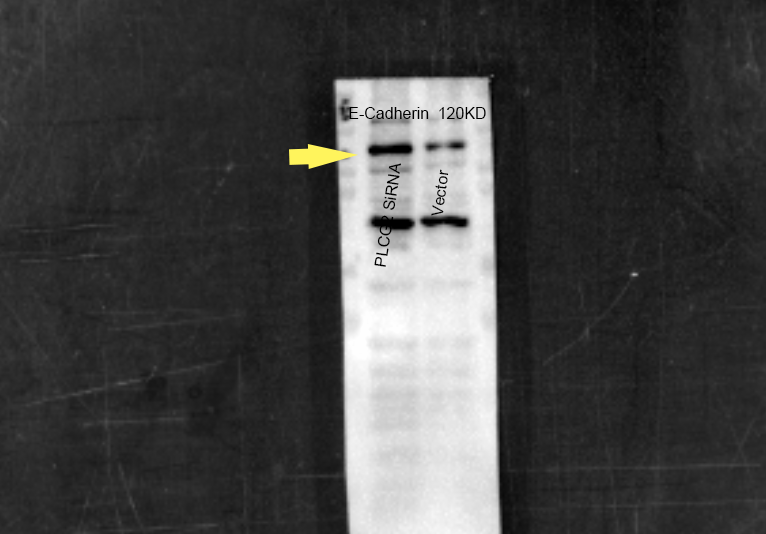


Fig4S E-Cadherin


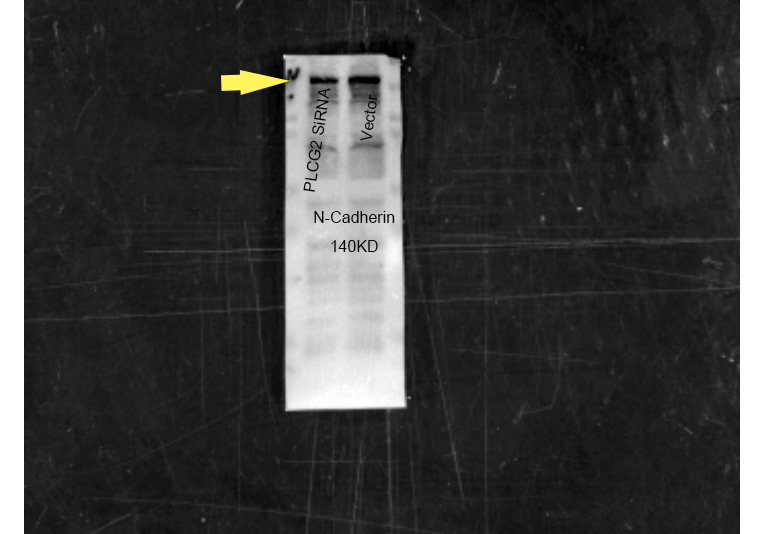


Fig4S N-Cadherin


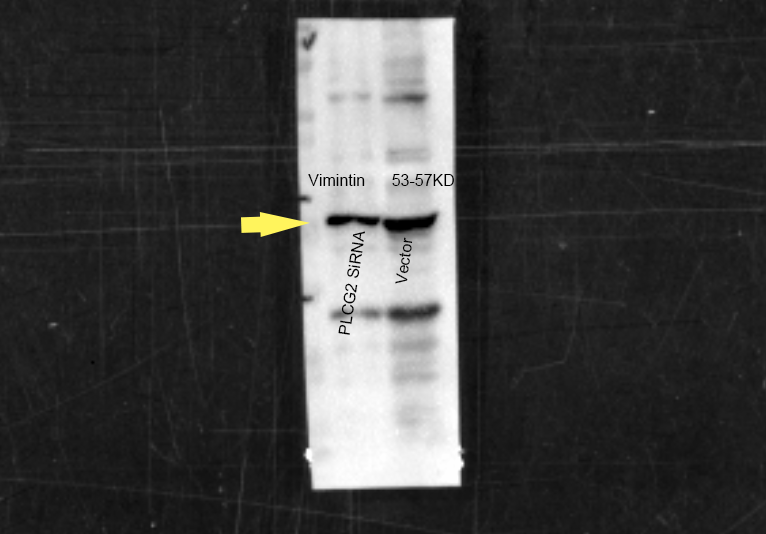


Fig4S Vimentin


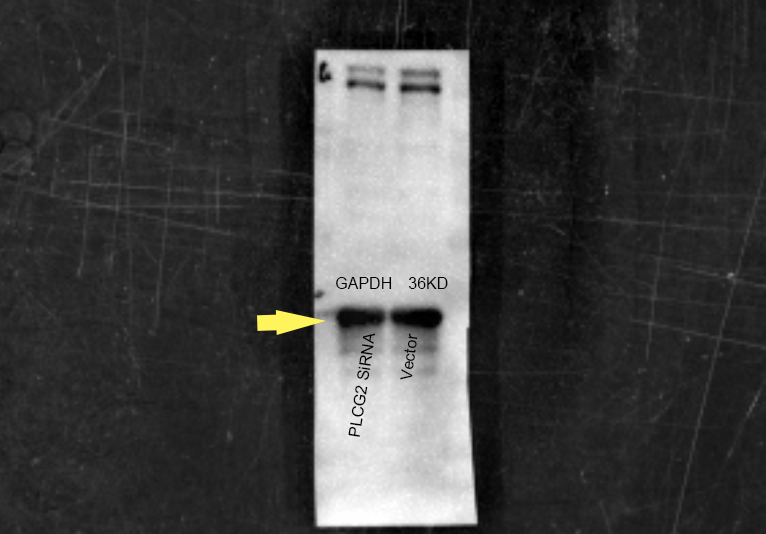


Fig5C GAPDH


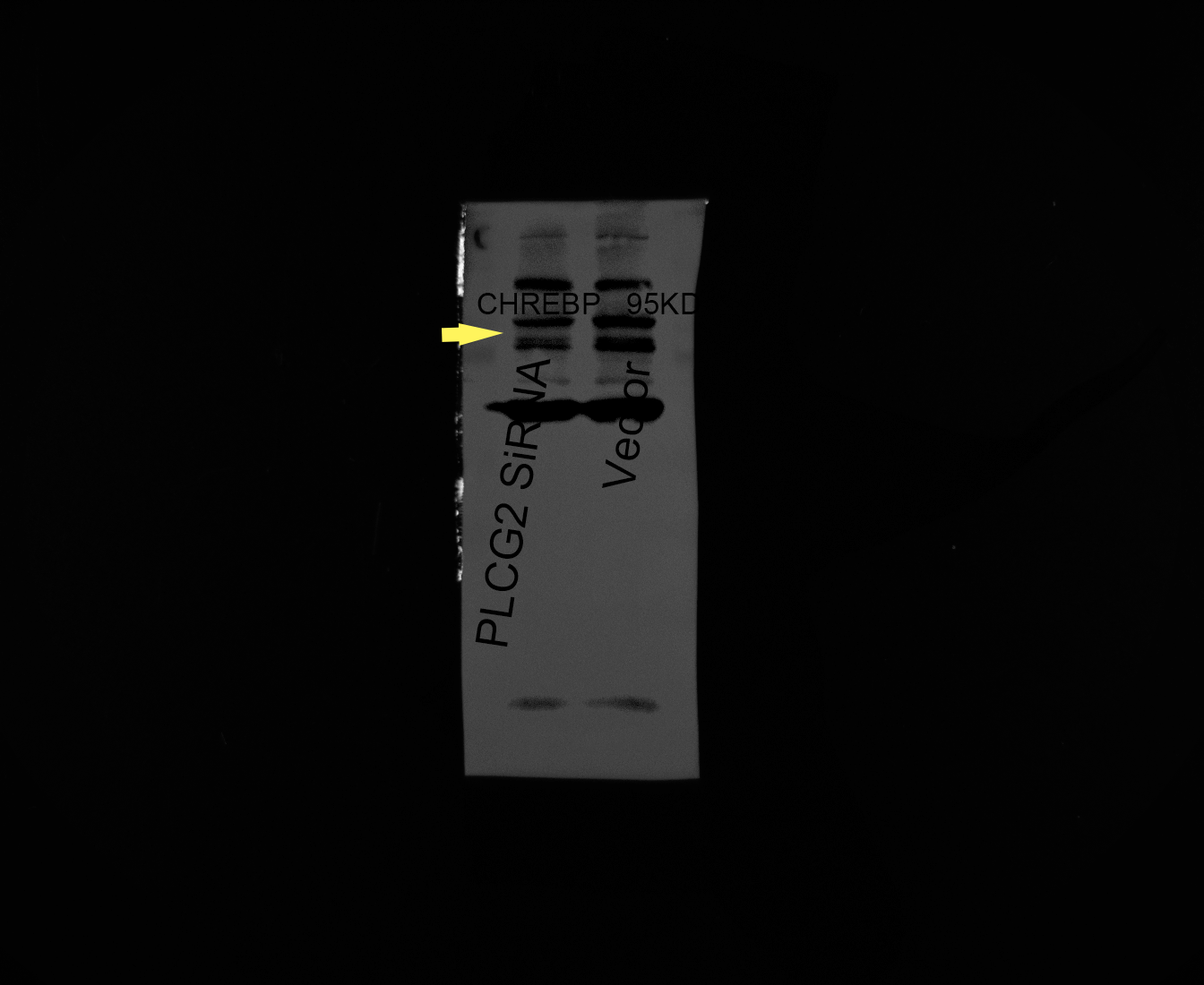


Fig5C CHREBP


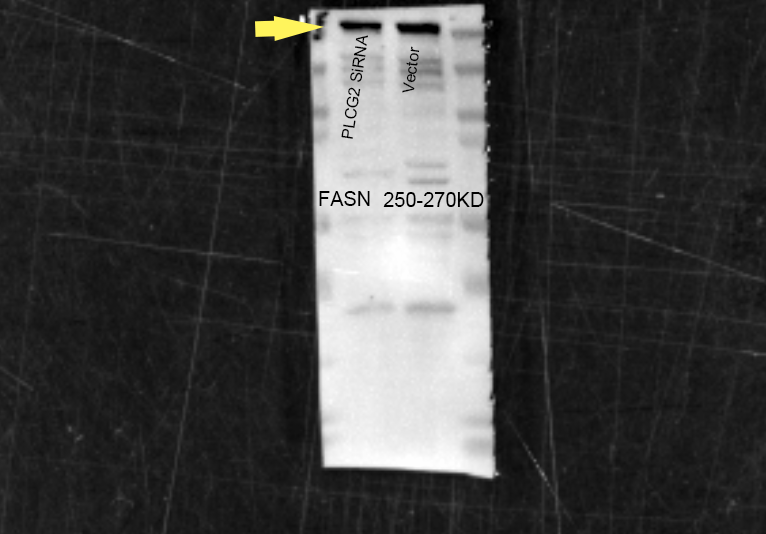


Fig5C FASN


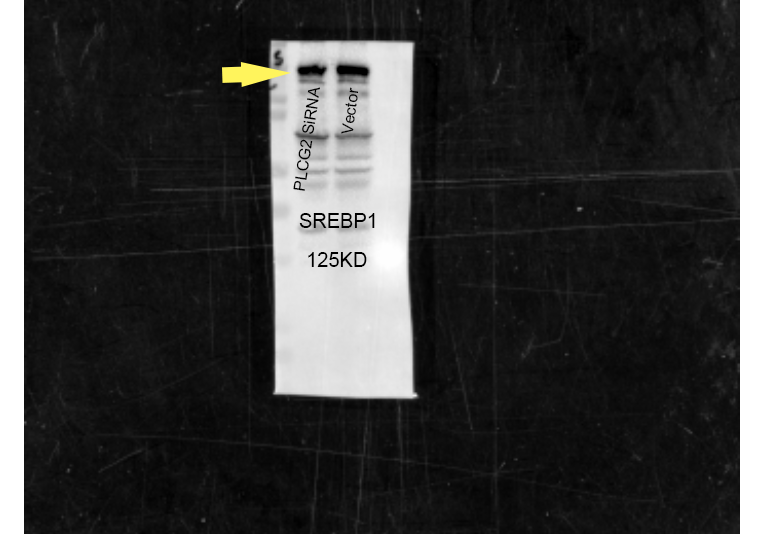


Fig5C SREBP1


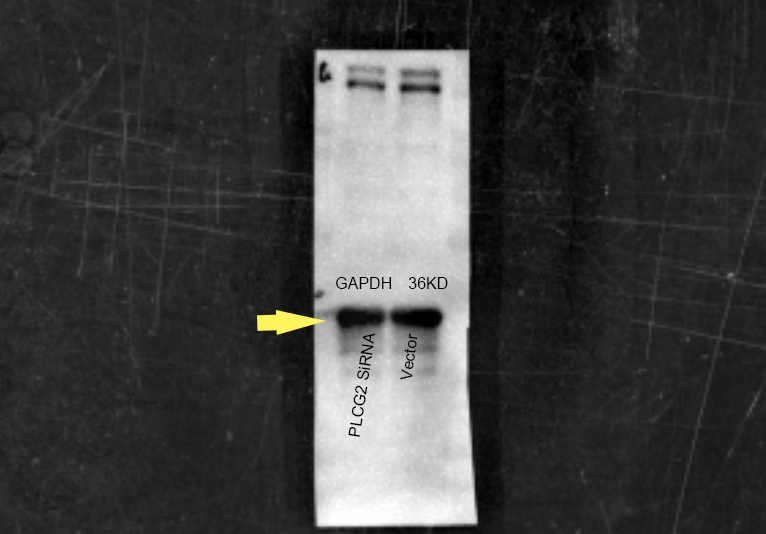


Fig5D GAPDH


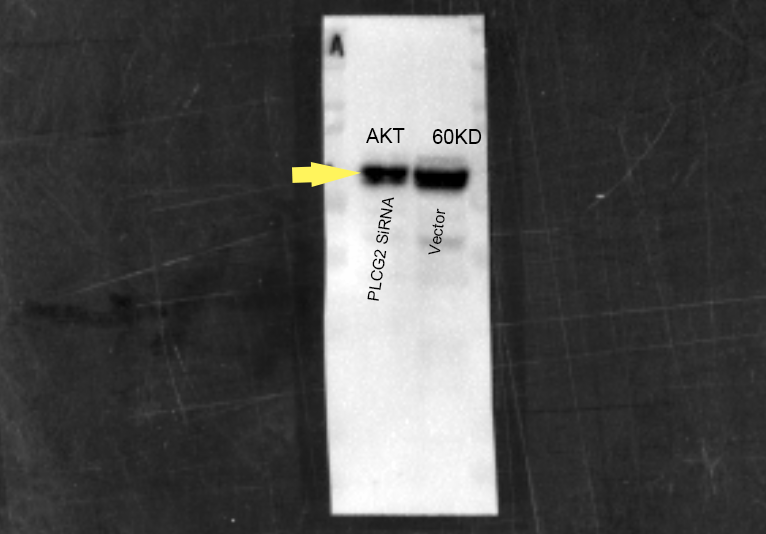


Fig5D AKT


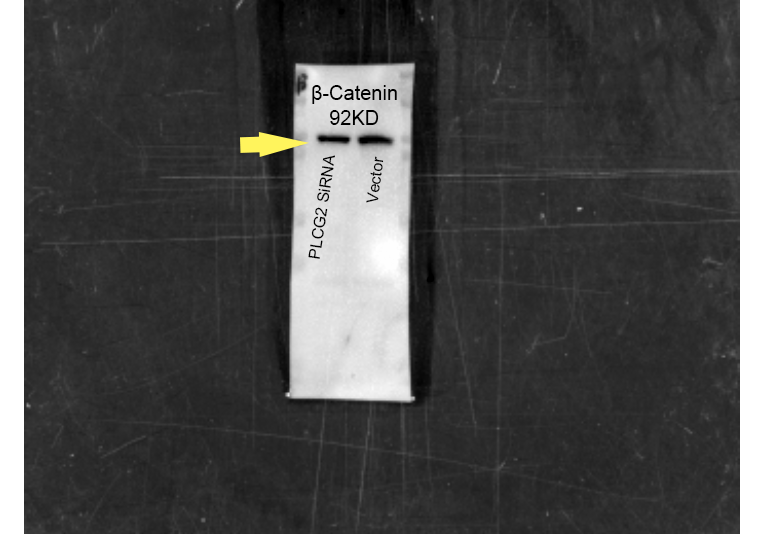


Fig5D β-Catenin


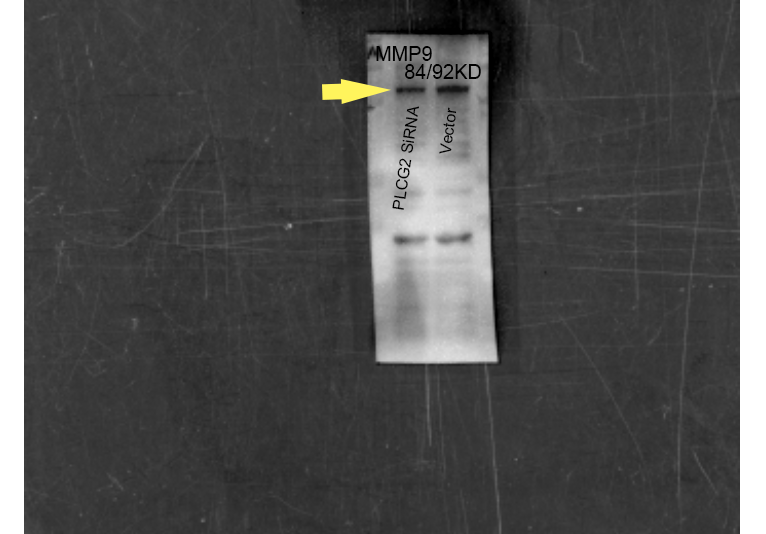


Fig5D MMP9


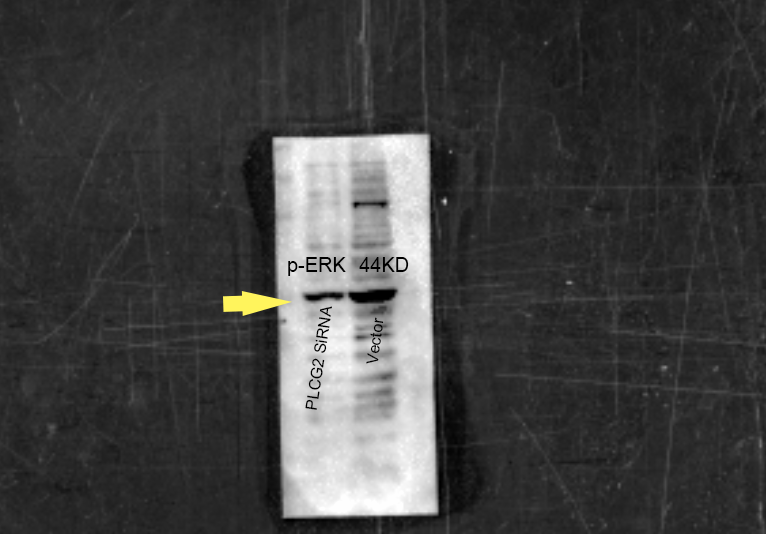


Fig5D p-ERK


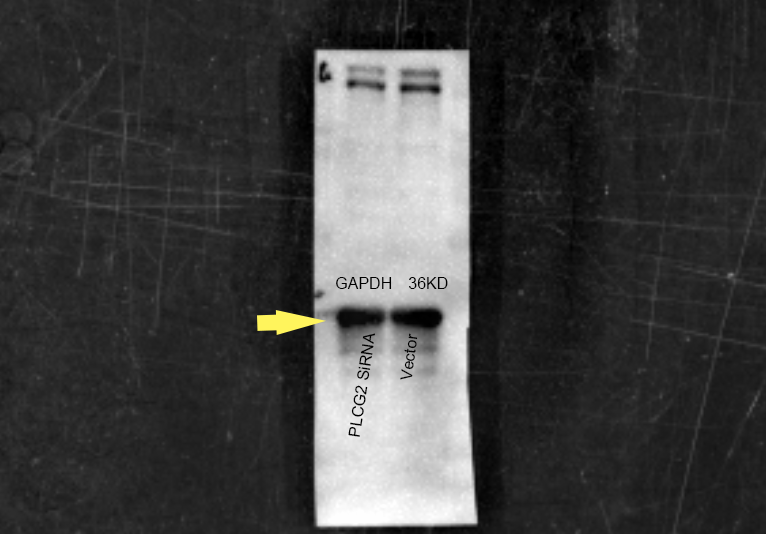


Fig5O GAPDH


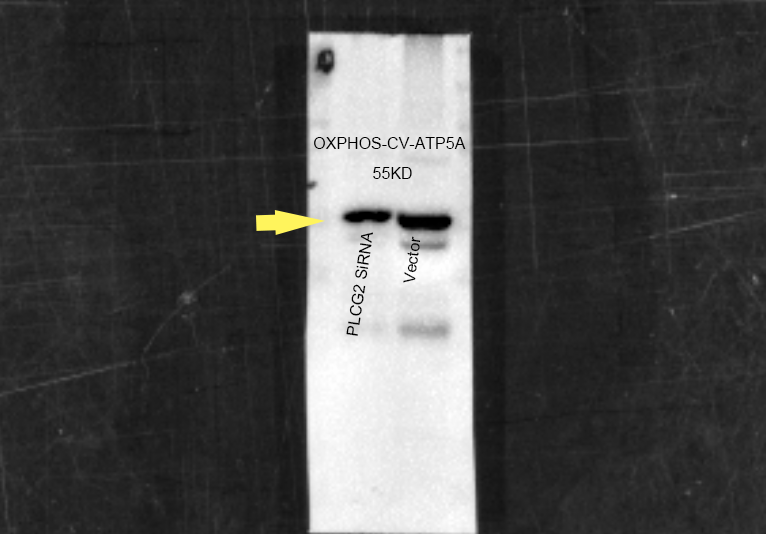


Fig5O-OXPHOS


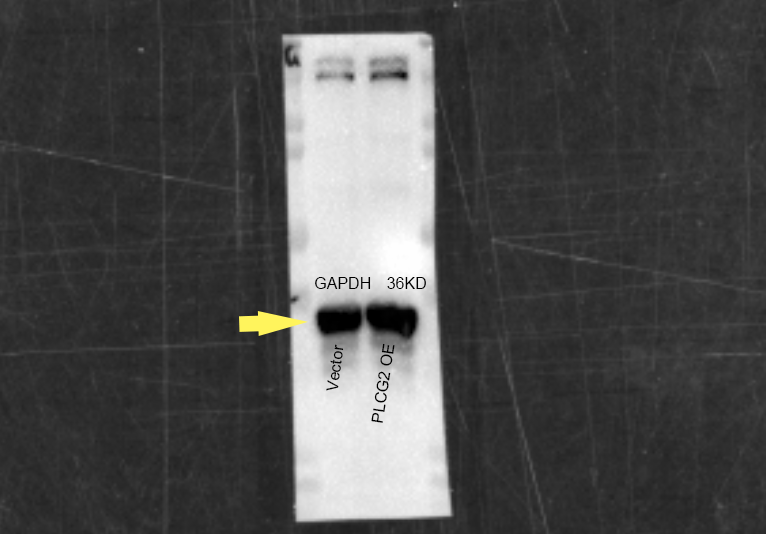


Fig6B GAPDH


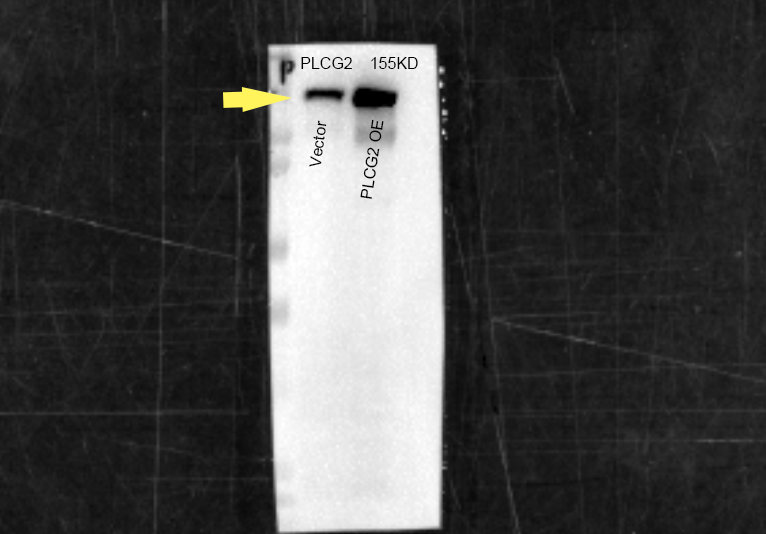


Fig6B PLCG2
